# Self‐Assembly of Unprotected Dipeptides into Hydrogels: Water‐Channels Make the Difference

**DOI:** 10.1002/cbic.202100518

**Published:** 2021-11-26

**Authors:** Ottavia Bellotto, Slavko Kralj, Michele Melchionna, Paolo Pengo, Matic Kisovec, Marjetka Podobnik, Rita De Zorzi, Silvia Marchesan

**Affiliations:** ^1^ Department of Chemical and Pharmaceutical Sciences University of Trieste Via L. Giorgieri 1 34127 Trieste Italy; ^2^ Materials Synthesis Department Jožef Stefan Institute Jamova 39 1000 Ljubljana Slovenia; ^3^ Department of Pharmaceutical Technology University of Ljubljana Aškerčeva 7 1000 Ljubljana Slovenia; ^4^ Unit of Trieste, INSTM Via L. Giorgieri 1 34127 Trieste Italy; ^5^ Department of Molecular Biology and Nanobiotechnology National Institute of Chemistry Hajdrihova 19 1001 Ljubljana Slovenia

**Keywords:** chirality, D-amino acids, hydrogels, peptides, self-assembly

## Abstract

Unprotected dipeptides are attractive building blocks for environmentally friendly hydrogel biomaterials by virtue of their low‐cost and ease of preparation. This work investigates the self‐assembling behaviour of the distinct stereoisomers of Ile‐Phe and Phe‐Ile in phosphate buffered saline (PBS) to form hydrogels, using transmission electron microscopy (TEM), attenuated total reflectance infrared spectroscopy (ATR‐IR), circular dichroism (CD), and oscillatory rheometry. Each peptide purity and identity was also confirmed by ^1^H‐ and ^13^C‐NMR spectroscopy and HPLC‐MS. Finally, single‐crystal XRD data allowed the key interactions responsible for the supramolecular packing into amphipathic layers or water‐channels to be revealed. The presence of the latter in the crystal structure is a distinctive feature of the only gelator of this work that self‐organizes into stable hydrogels, with fast kinetics and the highest elastic modulus amongst its structural isomers and stereoisomers.

## Graphical abstract

**The self‐assembling** behaviour of the distinct stereoisomers of Ile‐Phe and Phe‐Ile was studied in phosphate buffered saline (PBS) and found to form hydrogels. Single‐crystal XRD data of the four non‐enantiomeric dipeptides revealed water‐channels as a distinctive feature only for the heterochiral hydrogelator D‐Phe‐L‐Ile.

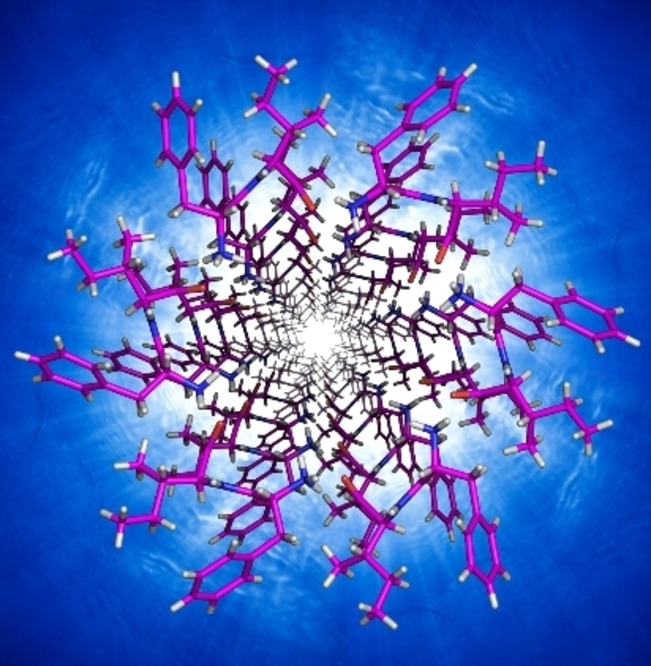

## Introduction

In recent years, there has been an increasing interest in the development of supramolecular hydrogels, especially for applications in medicine, owing to their ability to mimic natural tissues.^[1]^ Researchers have been studying a large diversity of components to tailor hydrogel properties, spanning from large biomolecules, such as proteins,^[2]^ DNA and nucleic acids,^[3]^ to synthetic polymers,^[4]^ nanoparticles,^[5]^ and carbon nanostructures.^[6]^ Among the various building blocks, minimalistic peptides have gained increasing attention in light of their ease of preparation and inherent biocompatibility, as well as the low‐cost of their production on a large scale.^[7]^ Whereas the majority of them exploit polyaromatic N‐capping groups to facilitate hydrophobicity‐driven self‐organization in water, there are still concerns over the biocompatibility of such N‐caps *in vivo*.^[8]^ To this end, the search is very active towards the discovery of N‐caps whose structure can promote self‐assembly and whose fate *in vivo* is benign.^[9]^ An alternative could be the use of short peptides with unprotected termini, yet the rules for their design to achieve hydrogels are still eluding scientists.^[10]^

In the case of dipeptides, those containing phenylalanine (Phe) are the obvious choice for self‐assembly, in light of the high‐propensity of this aromatic amino acid towards self‐organization in water.^[11]^ Phe‐Phe was recently reported to form metastable hydrogels.^[12]^ Interestingly, simple structural modifications were sufficient to stabilize the soft matter, for instance through dipeptide cyclization or Phe *p*‐substitution,^[12b]^ or change of stereoconfiguration into D‐Phe‐L‐Phe.^[13]^

Hydrophobicity is a well‐known factor to drive peptide self‐organization in water,^[10b]^ thus the search for unprotected dipeptides that gel could reasonably start from aliphatic amino acids. In particular, a sensible choice would fall on the most hydrophobic ones with butyl groups as the side chains, *i. e*., leucine (Leu) or isoleucine (Ile). In fact, removal of even just one methylene unit to yield the lesser hydrophobic valine (Val) could compromise self‐assembly. This hypothesis was confirmed by studies on Val‐Phe,^[14]^ and Phe‐Val,^[12b]^ that concluded the inability to self‐assemble of these dipeptides even at concentrations as high as 100 mM.

The case of Leu‐Phe and Phe‐Leu self‐assembly in water was recently described.^[15]^ Interestingly, both homochiral and heterochiral Leu‐Phe stereoisomers (*i. e*., L−L, D−L, L−D, or D−D) formed stable hydrogels at physiological conditions. On the contrary, only heterochiral Phe‐Leu (*i. e*., L−D, or D−L), but not their homochiral stereoisomers (*i. e*., L−L, or D−D) formed metastable hydrogels that rearranged into crystals in less than an hour. It was noted that stable gels arose from peptides that could establish both 1) extended hydrophilic interactions based on H‐bonding networks and N‐to‐C salt bridges between peptides, and 2) steric zippers, which are an established feature that stabilizes amyloids.^[16]^ The possibility that the presence of Phe at the C‐terminus could somehow favour hydrogelation was also considered, but the small dataset provided was not sufficient to verify this hypothesis, for which further examples were needed.

Surprisingly, thus far no study was reported on chirality effects on Ile‐Phe or Phe‐Ile, despite the fact that homochiral Ile‐Phe was observed to gel, although at concentrations as high as ca. 70 mM that required a small amount of organic solvent to aid its dissolution.^[14]^ This study thus aims at filling this gap, and verifying whether the self‐assembling trends observed for Leu‐Phe and Phe‐Leu stereoisomers apply also to their Ile‐containing counterparts. In this manner, our understanding of self‐assembling unprotected dipeptides could advance to assist with the future design of biomolecule‐based supramolecular hydrogels from simple and low‐cost building blocks.

## Results and Discussion

**Scheme 1:**
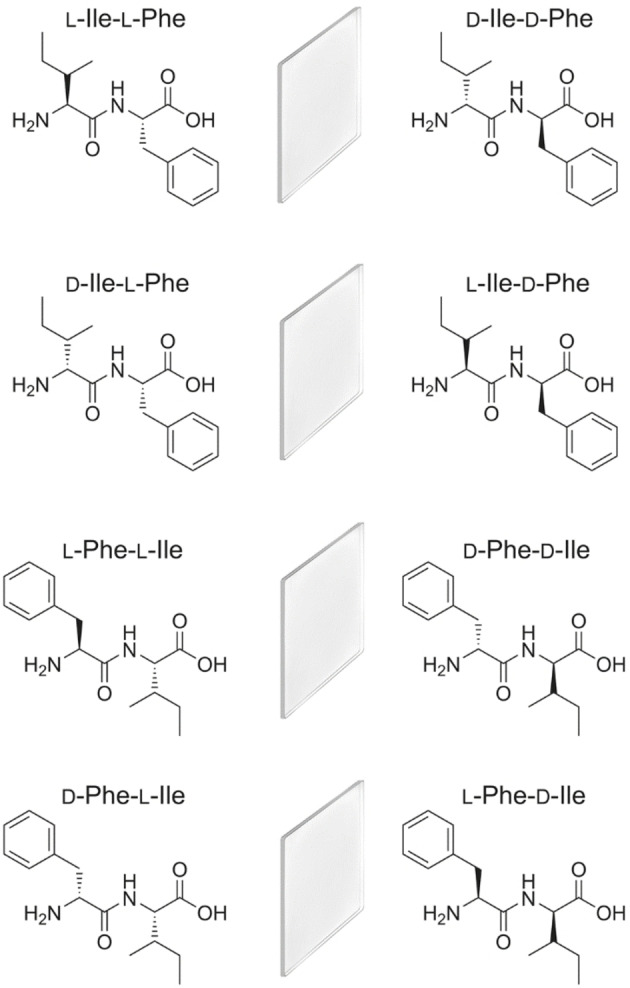
All possible stereo‐combinations of Ile and Phe in dipeptide sequences identify 8 compounds, which consist of 4 enantiomeric pairs, which are mirror‐image of each other (represented by a mirror between the two enantiomers).

If we consider all possible stereoconfigurations of Ile and Phe into a dipeptide sequence, we will obtain 8 possible compounds, *i. e*., L‐Ile‐L‐Phe, D‐Ile‐L‐Phe, L‐Ile‐D‐Phe, D‐Ile‐D‐Phe, L‐Phe‐L‐Ile, D‐Phe‐L‐Ile, L‐Phe‐D‐Ile, and D‐Phe‐D‐Ile (Scheme 1). Enantiomers have the same physicochemical properties, thus self‐assembling behaviour, in achiral environments, although they display different optical activity.^[17]^ It is worth to note that experimental works have sometimes reported differences, although these are likely ascribed to the level of purity across samples.^[18]^ This means that a smaller set, such as L‐Ile‐L‐Phe, D‐Ile‐L‐Phe, L‐Phe‐L‐Ile, and D‐Phe‐L‐Ile, will be representative also of the supramolecular behaviour in water of the mirror‐images (*i. e*., D‐Ile‐D‐Phe, L‐Ile‐D‐Phe, D‐Phe‐D‐Ile, and L‐Phe‐D‐Ile, respectively), thus allowing to understand the self‐assembly of all possible stereoconfigurations of Ile and Phe into dipeptides. Thus, in this study the supramolecular behaviour of the former four compounds was assessed to shed light on the sequences that gel in phosphate buffered saline and provide an initial screening for future studies.

### Synthesis and molecular characterization

The four compounds L‐Ile‐L‐Phe, D‐Ile‐L‐Phe, L‐Phe‐L‐Ile, and D‐Phe‐L‐Ile (shown on the left column of Scheme 1), were synthesized by solid‐phase using a standard Fmoc‐protection strategy, with HBTU/HOAt activation, and a final cleavage from the 2‐chlorotrytil resin in acidic conditions.^[19]^ The crudes were then purified by reversed‐phase HPLC and characterized by ^1^H‐NMR and ^13^C‐NMR spectroscopy, and LC‐MS as described in the Supporting Information. Interestingly, the HPLC retention times, which are an experimental measure of hydrophobicity,^[20]^ were as follows: 10.6 min (L‐Phe‐L‐Ile), 11.3 min (L‐Ile‐L‐Phe), 12.9 min (D‐Ile‐L‐Phe), and 13.0 min (D‐Phe‐L‐Ile). This data suggested that heterochirality increased peptide hydrophobicity, in agreement with previous observations.^[15]^ This could be the result of the spatial orientation of the side‐chains, which in the heterochiral compounds could be oriented on the same side of the peptide backbone, thus exposing the hydrophobic groups, and favouring the rise of amphipathic assemblies with net segregation between hydrophobic and hydrophilic components.^[21]^

### Self‐assembly into hydrogels

**Figure 1:**
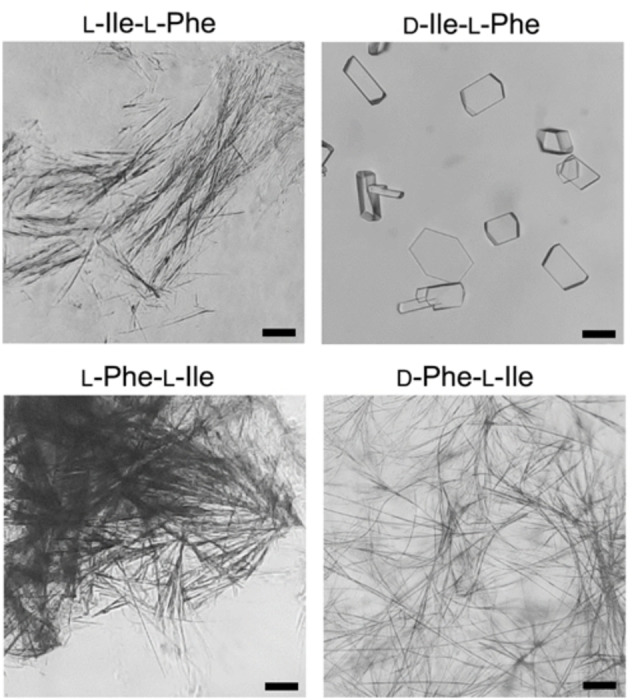
Optical microscopy images of anisotropic microstructures formed by peptides L‐Ile‐L‐Phe, D‐Ile‐L‐Phe, L‐Phe‐L‐Ile (at concentrations ≥40 mM), and D‐Phe‐L‐Ile (10 mM) in PBS. Scale bar=50 μm.

Hydrogelation was probed according to an established procedure, whereby the hydrophobic peptides were first dissolved in phosphate buffered saline (PBS, pH 7.3) with heating, and then samples were left to cool down to room temperature.^[15]^ Homochiral L‐Ile‐L‐Phe and L‐Phe‐L‐Ile yielded solutions even at concentrations as high as 70 mM, although at 50 mM or above it was possible to see the presence of rare instances of microfibers floating in solution, suggesting a tendency to fibrillate (see Figure 1). However, the more hydrophobic compound of the two, i. e., L‐Ile‐L‐Phe, was previously reported to gel at concentrations of 50–70 mM, but in a different solvent system.^[14]^ For this reason, here it was tested up to 100 mM in PBS, yet no gel was formed. Rheology confirmed the presence of a solution (G’<G”), with an increase of the viscous component G” over the first 10 minutes (see Supporting Information), which was ascribed to the presence of fibrils.

**Figure 2:**
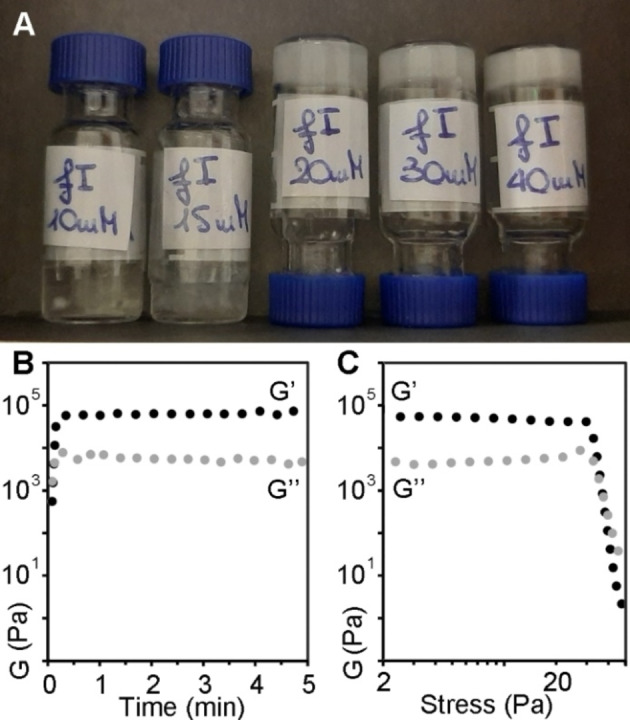
A) Hydrogels of D‐Phe‐L‐Ile at 20 mM (mgc, centre) or above (30 mM or 40 mM, right). At lower concentrations (10 or 15 mM, left), heterogenous systems contained both liquid and solid phases. B–C) Rheology data confirmed the immediate gelation of D‐Phe‐L‐Ile at 40 mM, reaching a plateau within one minute (B), and stress sweeps (C) showed a gel‐to‐sol transition at ca. 46 Pa.

Different was the outcome in the case of the two more hydrophobic, heterochiral analogues. Increasing the concentration of D‐Ile‐L‐Phe to 40 mM and above resulted in the formation of crystals (Figure 1, top right), which were suitable for X‐ray diffraction analysis (*vide infra*), while D‐Phe‐L‐Ile yielded microfibers already at 10 mM (Figure 1, bottom right), and an opaque hydrogel with a minimum gelling concentration (mgc) of 20 mM (Figure 2A). Oscillatory rheology was thus employed to confirm visual observations and to characterize the hydrogel formed by D‐Phe‐L‐Ile (Figure 2B–C). To allow for a meaningful comparison with the literature data on Leu‐Phe and Phe‐Leu isomers, the concentration of 40 mM was chosen for the tests. No hydrogel was formed for any of the other three compounds (*i. e*., L‐Ile‐L‐Phe, D‐Ile‐L‐Phe, and L‐Phe‐L‐Ile). Using 10 mM PBS or deionized water adjusted to the same pH led to analogous results (see Supporting Information). In contrast, D‐Phe‐L‐Ile gelled at all conditions within 20 seconds, also when residual trifluoroacetate was exchanged to chloride (see Supporting Information). In PBS, it reached an elastic modulus of ca. 7.0×10^4^ Pa at plateau within minutes (Figure 2B). Stress sweeps revealed a gel‐to‐sol transition occurring at 46 Pa (Figure 2C). Frequency sweeps confirmed the hydrogel nature, with the elastic modulus (G’) higher than the viscous modulus (G”), with both being independent from the applied frequency (see Supporting Information). For comparison, its structural isomer D‐Phe‐L‐Leu under analogous experimental conditions (40 mM in PBS) yielded a metastable hydrogel with an elastic modulus of 1 kPa that disassembled within minutes, thus suggesting that D‐ Phe‐L‐Ile is a better gelator than its structural isomer D‐Phe‐L‐Leu.^[15]^ The other gelling isomers L‐Leu‐L‐Phe and D‐Leu‐L‐Phe formed stable hydrogels, although with much slower kinetics relative to D‐Phe‐L‐Ile, and with significantly lower elastic modulus G’ of 10 kPa.^[15]^

### Transmission electron microscopy (TEM) analysis

**Figure 3:**
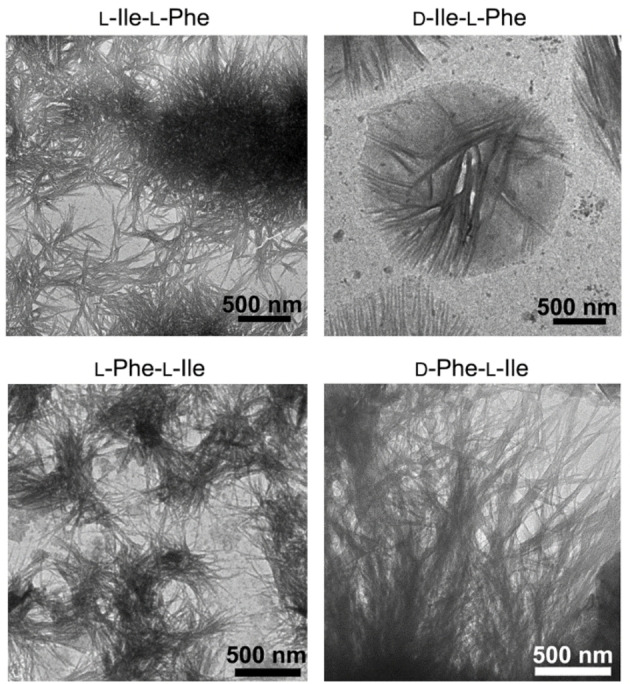
TEM micrographs of the four compounds (40 mM). Nanofibrils were present in all samples, although only in the case of gelling D‐Phe‐L‐Ile (bottom right) they reached several microns in length. In all the other cases, clusters of shorter fibrils were mainly present, with recurrent sub‐micron lengths.

TEM micrographs were acquired to shed light on the ability of each of the four dipeptides to self‐organize into nanostructures. Surprisingly, nanofibrils were observed in all cases (Figure 3), despite their diverse ability to form hydrogels. There was no significant difference in terms of average fibril diameter across samples (11.6±4.9 nm, 11.9±2.1 nm, 11.6±2.9 nm, and 11.8±2.9 nm, for L‐Ile‐L‐Phe, D‐Ile‐L‐Phe, L‐Phe‐L‐Ile, and D‐Phe‐L‐Ile, respectively, with *n*=100). Compared to gelling dipeptides L‐Leu‐L‐Phe and D‐Leu‐L‐Phe,^[15]^ fibrils were significantly thinner, in agreement with previous observations that the β‐branching on the side chain of Ile, near the peptide backbone, disfavours fibril bundling compared to analogues bearing the γ‐branched Leu.^[22]^ Furthermore, in the case of D‐Ile‐L‐Phe, also microcrystals were found (see Supporting Information), besides the nanofibrils, in agreement with optical microscopy imaging. It was evident that only in the case of gelling D‐Phe‐L‐Ile, fibrils consistently exceeded several microns in length, effectively creating an interconnected network. In all the other cases, fibrils were rarely longer than 1–2 microns, thus yielding isolated clusters with limited interconnectivity (Figure 3). These observations were in agreement with the gelation ability noted only for D‐Phe‐L‐Ile in PBS, and we inferred a similar supramolecular organization for all isomers at the nano‐scale, with only minor differences that were amplified at the micro‐scale.

We also performed cryoTEM (see Supporting Information, Figure S33), since concerns exist over the possibility of artefacts arising from the drying step required for TEM analysis, despite the fact that previous studies on similar peptides demonstrated correspondence between cryoTEM and TEM micrographs.[^22^, ^23^] Unfortunately, we could image successfully only microcrystals of D‐Phe‐L‐Ile (Figure S33D).

### Spectroscopic analysis of peptide conformations

Each compound was also characterized by circular dichroism (CD) and attenuated‐total‐reflectance infrared (ATR‐IR) spectroscopies to shed light on peptide conformations leading to the observed nanostructures. CD offers useful information regarding the spatial arrangement of chiral molecules. In the case of longer peptides, it is widely applied to determine secondary conformations, such as α‐helices, β‐sheets, *etc*., which display characteristic signatures. Dipeptides, such as those studied herein, are too short as sequences to satisfy the requirements that define such conformations. However, also single amino acids display CD spectra, which are positive in the 200–250 nm region for those with L‐stereocofiguration.^[24]^ Therefore, their D‐enantiomers will display mirror‐imaged negative spectra in the same region. Yet, the case of heterochiral dipeptides is far more complex to predict. While some studies reported that the stereoconfiguration of the amino acids at the peptide termini dictated the sign of the CD spectrum,^[25]^ others showed that multiple factors come into play, such as solvent.^[26]^

**Figure 4:**
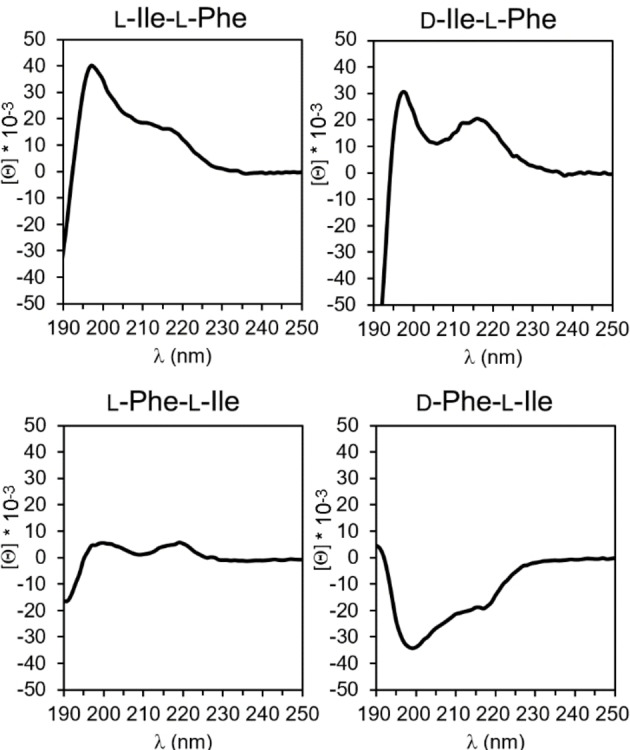
CD spectra of the four compounds.

Interestingly, the CD spectra of the four dipeptides studied herein (Figure 4) were strikingly similar to those of their analogues with Leu in place of Ile.^[15]^ In both cases, the only negative spectrum was the one of the dipeptide bearing D‐Phe at the N‐terminal. Since enantiomers are expected to display mirror‐imaged CD spectra, we can conclude that in the small library of 16 dipeptides that arise from the combination of Phe with either Leu or Ile (*i. e*., the 8 sequences shown in Scheme 1 and the corresponding 8 analogues with Leu in place of Ile), it is the stereoconfiguration of Phe that dictates the sign of the CD spectrum, irrespective of its position along the sequence (*i. e*., at the N‐ or C‐terminus of the dipeptide). The spectra, characterized by maxima (for L‐Ile‐L‐Phe, D‐Ile‐L‐Phe, and L‐Phe‐L‐Ile) or minima (for D‐Phe‐L‐Ile) at 198 and 218 nm, were reminiscent of the signatures reported for the self‐assembling L‐Phe‐D‐Ile‐L‐Phe.^[22]^ In that case, a thorough experimental and *in silico* investigation allowed to decipher it as indication of a population of conformations, of which the most visited ones featured dihedral angles typical of β‐structures (sheets and turns).^[22]^ We thus inferred a similar case here, as confirmed by dihedral angles obtained from the crystal structures (see Supplementary Information, Table S2).

**Figure 5:**
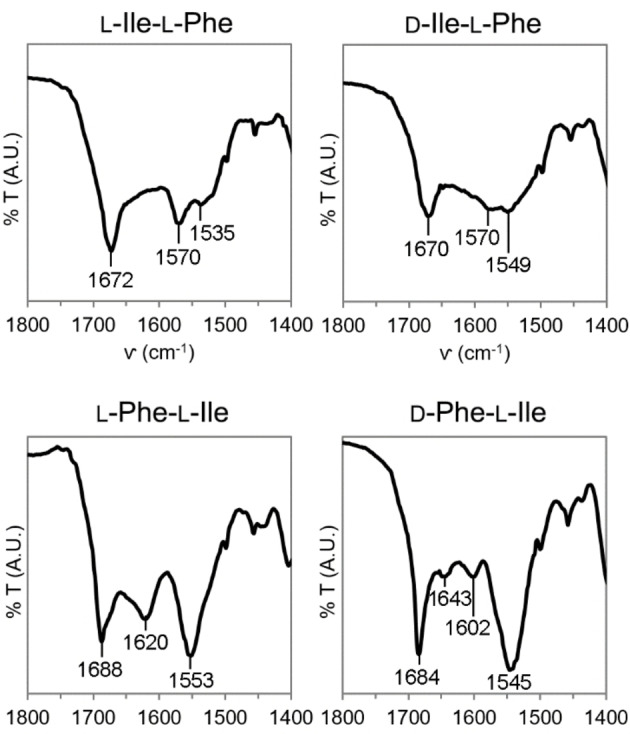
Amide I region of ATR‐IR spectra of the four compounds.

Dipeptide conformation was also probed by ATR‐IR. The amide region (Figure 5) is indicative of the hydrogen bonding pattern, hence, for longer peptides, of their conformation. All spectra featured a characteristic peak in the 1670–1680 cm^−1^ range, where β‐turns are located for longer sequences.^[27]^ Another signal, which was clearly visible only for Phe‐Ile stereoisomers, occurred in the β‐structure range (1600–1620 cm^−1^). The amide II featured a peak at 1570 cm^−1^ for Ile‐Phe stereoisomers, and another one at 1535 cm^−1^ for L‐Ile‐L‐Phe, which was shifted to 1545–1553 cm^−1^ for the other three analogues. The signal at 1570 cm^−1^ was already reported for L‐Ile‐L‐Phe, and it had been attributed to the strong association between the charged peptide termini in the supramolecular state.^[14]^

### Single‐crystal X‐ray diffraction analysis

**Figure 6:**
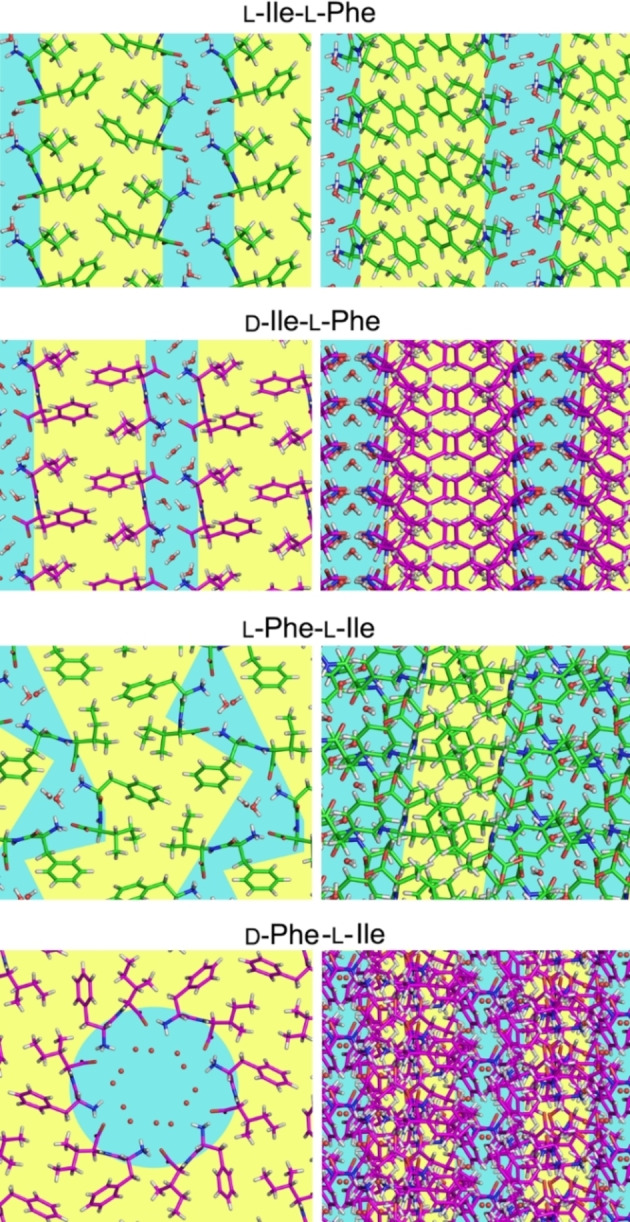
Single‐crystal XRD structures of L‐Ile‐L‐Phe,^[28]^ D‐Ile‐L‐Phe (CCDC 109139), L‐Phe‐L‐Ile,^[29]^ and D‐Phe‐L‐Ile (CCDC 109138). The first three dipeptides formed parallel stacks (top‐view on the left, and side‐view on the right) that defined amphipathic layers (hydrophilic regions with solvent molecules are shown in light blue, and hydrophobic areas in yellow). The only PBS gelator D‐Phe‐L‐Ile formed instead amphipathic water channels whose cavity was defined by six zwitterionic molecules arranged head‐to‐tail.

The ionic interactions between charged termini were confirmed by single‐crystal XRD analysis (Figure 6). The structures of the homochiral dipeptides L‐Ile‐L‐Phe^[28]^ and L‐Phe‐L‐Ile^[29]^ were already described in the literature, while those of D‐Ile‐L‐Phe and D‐Phe‐L‐Ile were solved in this work. We also attempted the crystallization of the homochiral compounds, with no success.

In all cases of non‐gelling dipeptides, molecules self‐organized into amphiphilic layers that consisted of parallel stacks with an extended H‐bonding network. However, water molecules bridged peptide‐peptide interactions along the stack through H‐bonds. In particular, L‐Phe‐L‐Ile displayed the most complex packing of the three isomers, with two independent dipeptide molecules in the cell unit, each with a very different conformation (see Supplementary Information, Table S2).^[29]^ By contrast, for l‐ile‐L‐Phe,^[28]^ and D‐Ile‐L‐Phe, just one independent dipeptide molecule was present in the cell unit. Moreover, only in the former instance there was an evident steric zipper defined by the aromatic rings, and this is a well‐known stabilizing feature for amyloid structures^[16]^ that could justify its ability to gel in different conditions^[14]^ than those probed here.

Remarkably, only the gelator D‐Phe‐L‐Ile displayed a supramolecular arrangement analogous to Phe‐Phe,^[13]^ with six peptide zwitterions arranged head‐to‐tail so as to define an amphipathic water channel, with a hydrophilic interior and hydrophobic exterior, with a clear separation between hydrophilic and hydrophobic regions (Figure 6, bottom). In particular, peptides arranged in a tubular fashion along the *c* crystallographic direction, through H‐bonds between amides. Interestingly, molecules forming the walls of the tube are related by the 3‐fold crystallographic axis, a symmetry element belonging to the *R3* space group of the crystal, but also by a 6‐fold non‐crystallographic symmetry that relates the two independent peptide molecules present in the unit cell, and two of the independent water molecules with the other two (see Supporting Information). Tubes held together by hydrophilic interactions interact with each other through weaker intermolecular and intramolecular CH‐π interactions, between the Phe and Ile side‐chains (see Supporting Information).

## Conclusion

In conclusion, this work reported D‐Phe‐L‐Ile as a new hydrogelator in PBS with mgc of 20 mM, and with the ability to self‐organize into amphipathic water channels. Interestingly, the other stereo‐ and regio‐isomers did not gel in PBS at concentrations as high as 70 mM, despite their ability to self‐organize into anisotropic nanostructures as confirmed by microscopy. In particular, although all compounds could form fibrils of analogous diameter, their length varied, with only the gelling D‐Phe‐L‐Ile being able to form several‐micron long fibrils that effectively entangled in a three‐dimensional network that yielded a macroscopic hydrogel.

Comparison with the analogous dipeptide series containing Leu and Phe,^[15]^ revealed that heterochirality overall increased hydrophobicity, which promoted self‐assembly, although this was not sufficient as a parameter on its own for hydrogelation to occur. All the gelling dipeptides showed the presence of recurrent packing features, *i. e*., 1) the ability to segregate hydrophilic and hydrophobic components into separate areas, with polar solvent molecules being confined in the former; 2) the establishment of an extended network of H‐bonds between amides of adjacent dipeptides, as well as salt bridges between charged termini; 3) the ability to form steric zippers especially with Phe side‐chains that is a well‐known amyloid‐stabilizing feature.

Overall, it appears evident that the supramolecular behaviour and gelling ability of simple biomolecules such as unprotected dipeptides is difficult to predict *a priori*, yet recurrent features in terms of the supramolecular packing of gelators are being identified as research in the field advances. The emergence of simple design rules for their self‐organization into soft matter will ultimately accelerate developments for finding applications in a variety of fields, spanning from nanotechnology and smart materials to innovative therapeutics. Ultimately, they may shed further light on prebiotic chemistry and the origins of life.

## Experimental Section

### Materials and methods

Fmoc protected amino acids, 2‐chlorotrityl resin, *O*‐benzotriazole‐*N,N,N,N*‐tetramethyl‐uronium‐hexafluoro‐phosphate (HBTU), and 1‐hydroxy‐7‐azabenzotriazole (HOAt) were purchased from GL Biochem (Shanghai) Ltd. All solvents and the other chemicals were purchased from Merck, at analytical grade. High purity Milli‐Q‐water with a resistivity greater than 18 M Ω cm was obtained from an in‐line Millipore RiOs/Origin system. ^1^H‐NMR and ^13^C‐NMR spectra were recorded at 400 MHz on a Varian Innova Instrument with chemical shift reported as ppm (with tetramethylsilane as internal standard). ESI‐MS spectra were recorded on an Agilent 6120 single quadrupole LC‐MS system. Optical microscope images were acquired on a Leitz Labovert instrument with a 20× magnification objective on a drop of fresh samples deposited on a clean glass slide.

### Dipeptide synthesis, purification, and self‐assembly

Each dipeptide was synthesised by standard solid‐phase methods and purified by reversed‐phase HPLC as described previously.^[19]^ The method used consisted of a mixture of MeCN/water with 0.05 % trifluoroacetic acid, 3 ml/min, solvent gradient: 0–3 min 25 % MeCN, 16 min 95 % MeCN using a C‐18 column (Kinetex, 5 μm, 100 Å, 250×10 mm, Phenomenex). Each compound was dissolved in phosphate‐buffered saline solution at the desired concentration with the aid of an ultrasound bath (Branson 500) for a few seconds, followed by vial immersion in an oil bath at 100 °C until a clear solution was obtained (25–30 min.). Samples were then left to cool down to room temperature (1 min).

### Oscillatory rheology

Dynamic time sweep rheological analyses were carried out on a Malvern Kinexus Ultra Plus Rheometer (Alfatest, Milan, Italy) with a 20 mm stainless steel parallel plate geometry. The temperature was maintained at 25 °C using a Peltier temperature controller. Samples were prepared in situ and immediately analysed with a gap of 1 mm. Time sweeps were recorded at 1 Hz and 1 Pa. Frequency sweeps were recorded at 1 Pa, and then stress sweeps were recorded using a frequency of 1 Hz.

### Transmission electron microscopy (TEM) imaging

Dipeptide nanostructures were visualized with a Jeol JEM 2100 instrument at 100 kV. Briefly, 5 μL of dipeptide samples were transferred with a micropipette onto a carbon‐coated 300‐mesh grid, which was previously exposed for 6 min. under ultraviolet (UV) ozone cleaner just before material deposition. After 1 min of adsorption, the excess material was drawn off, and 5 μL of a 2 % aqueous potassium phosphotungstate at pH 7.2 was poured on the grids. Samples were then dried in a desiccator *in vacuo* prior to imaging.

### Circular dichroism (CD) spectroscopy

A 0.1 mm quartz cell was used on a Jasco J815 Spectropolarimeter, with 1 s integrations, 1 accumulation and a step size of 1 nm with a bandwidth of 1 nm at 25 °C. Samples were prepared at a peptide concentration of 1 mM in milliQ water and the pH was adjusted to neutral with NaOH. Shown spectra are the average of at least 5 measurements.

### Attenuated total reflectance (ATR) infrared spectroscopy

The Infrared (IR) spectra were recorded with an Affinity‐1S Shimadzu FT‐IR, equipped with a QATR accessory, diamond crystal. A drop of the hydrogel was placed on a silicon wafer, and then dried under vacuum overnight. Spectra were acquired with 240 accumulations and 4 cm^−1^ resolution and those shown are the average of at least 3 measurements.

### Single‐crystal X‐ray diffraction (XRD)

Single crystals of D‐Ile‐L‐Phe (CCDC 109139) were obtained after approximately 1 hour of sample preparation as described above (40 mM in PBS). Single crystals of D‐Phe‐L‐Ile (CCDC 109138) were obtained under the same conditions after a month from the corresponding hydrogel. For each compound, a single crystal was collected with a loop, cryoprotected by dipping the crystal in glycerol, and stored frozen in liquid nitrogen. The crystal was mounted on the diffractometer at the synchrotron Elettra, Trieste (Italy), beamline XRD1, using the robot present at the facility. Temperature was kept at 100 K. Diffraction data were collected by the rotating crystal method using synchrotron radiation, wavelength 0.70 Å. Further details on structure determination and cell unit parameters are provided in the Supplementary Information.

Deposition Numbers 109139 and 109138 refer to thecrystallographic data deposited at the Cambridge Crystallographic Data Centre (CCDC).

## Conflict of interest

The authors declare no conflict of interest.

## Supporting information

As a service to our authors and readers, this journal provides supporting information supplied by the authors. Such materials are peer reviewed and may be re‐organized for online delivery, but are not copy‐edited or typeset. Technical support issues arising from supporting information (other than missing files) should be addressed to the authors.

Supporting Information
