# Supplementary material for: Self‐Assembly of Unprotected Dipeptides into Hydrogels: Water‐Channels Make the Difference
Source: Chembiochem. 2021 Nov 26;23(2):e202100518. doi: 10.1002/cbic.202100518 (PMC9299199; doi:10.1002/cbic.202100518)
Supplement: Supplementary file 1 — Supporting Information [file CBIC-23-0-s001.pdf]

ChemBioChem

Supporting Information

Self-Assembly of Unprotected Dipeptides into Hydrogels: Water-Channels Make the Difference

Ottavia Bellotto, Slavko Kralj, Michele Melchionna, Paolo Pengo, Matic Kisovec, Marjetka Podobnik, Rita De Zorzi, and Silvia Marchesan*

Supporting Information

Table of Contents

1. L-Ile-L-Phe spectroscopic data………………………..……………………… 2
2. D-Ile-L-Phe spectroscopic data………………………..……………………… 5
3. L-Phe-L-Ile spectroscopic data……………………...………………………… 8
4. D-Phe-L-Ile spectroscopic data.………………..………………………………... 11
5. HPLC traces………………………………………………………………………. 14
6. Rheology data for D-Phe-L-Ile hydrogel……………….……………….……...... 14
7. Rheology data for L-Ile-L-Phe (40 mM) viscous solution in PBS…………… 15
8. Single-crystal XRD data…………..……………………………………………... 15
9. High-resolution transmission electron microscopy (HR-TEM) data…………. 22
10. CryoTEM imaging data…………………………………………………………….. 22
11. ^{19}F-NMR, TFA removal and self-assembly with chloride counterion………. 23
12. Self-assembly in 10 mM PBS and deionized water…………………………... 24

1. L-Ile-L-Phe spectroscopic data

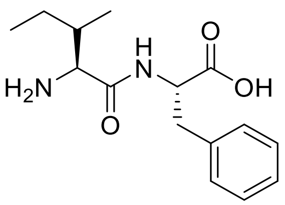

^{1}H NMR (400 MHz, DMSO-d_6, TMS), δ (ppm): 8.66 (d, J = 8.0 Hz, 1H, NH), 7.32 – 7.19 (m, 5H, Ar), 4.48 (ddd, J = 8.0, 8.0, 5.2 Hz, 1H, αCH Phe), 3.62 (d, J = 5.2 Hz, 1H, αCH Ile), 3.09 (dd, J = 14.4, 5.2 Hz, 1H, βCH_2 Phe), 2.95 (dd, J = 14.4, 8.0 Hz, 1H, βCH_2 Phe), 1.90 – 1.77 (m, 1H, βCH Ile), 1.44 (ddddd, J = 14.8, 7.4, 7.4, 7.4, 3.6 Hz, 1H, γCH_2), 1.08 (m, 1H, γCH_2), 0.92 (d, J = 6.8 Hz, 3H, γCH_3), 0.84 (dd, J = 7.4, 7.4 Hz, 3H, δCH_3). **^{13}C NMR** (100 MHz, DMSO-d_6, TMS), δ (ppm): 172.3, 168.2 (2 x CO); 137.2, 129.1, 128.3, 126.6 (Ar); 56.5, 53.8 (2 x αC); 36.5, 36.4 (2 x βC); 23.4, 14.6 (2 x γC); 11.2 (1 δC). MS (ESI): m/z 279.1 $(M+H)^+$, 301.1 $(M+Na)^+$; 277.1 $(M-H)^-$.

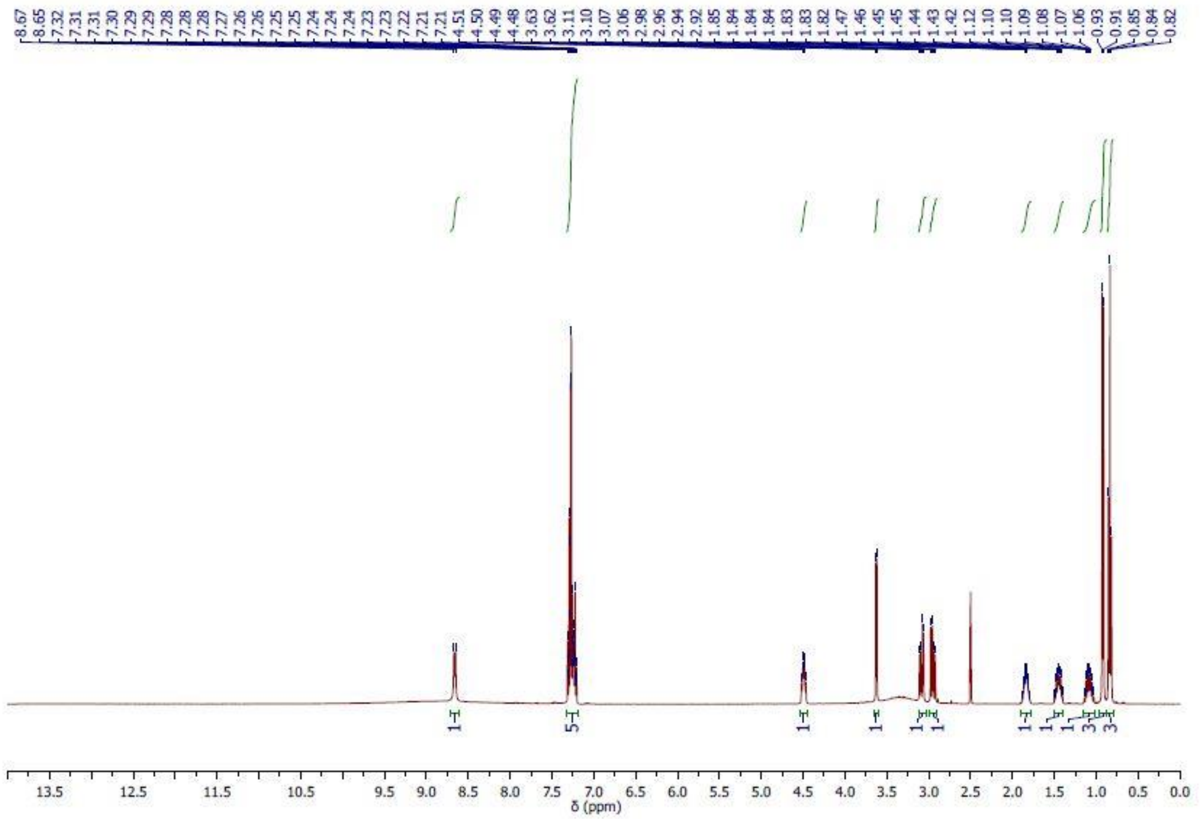

Fig. S1. ^{1}H-NMR spectrum of L-Ile-L-Phe.

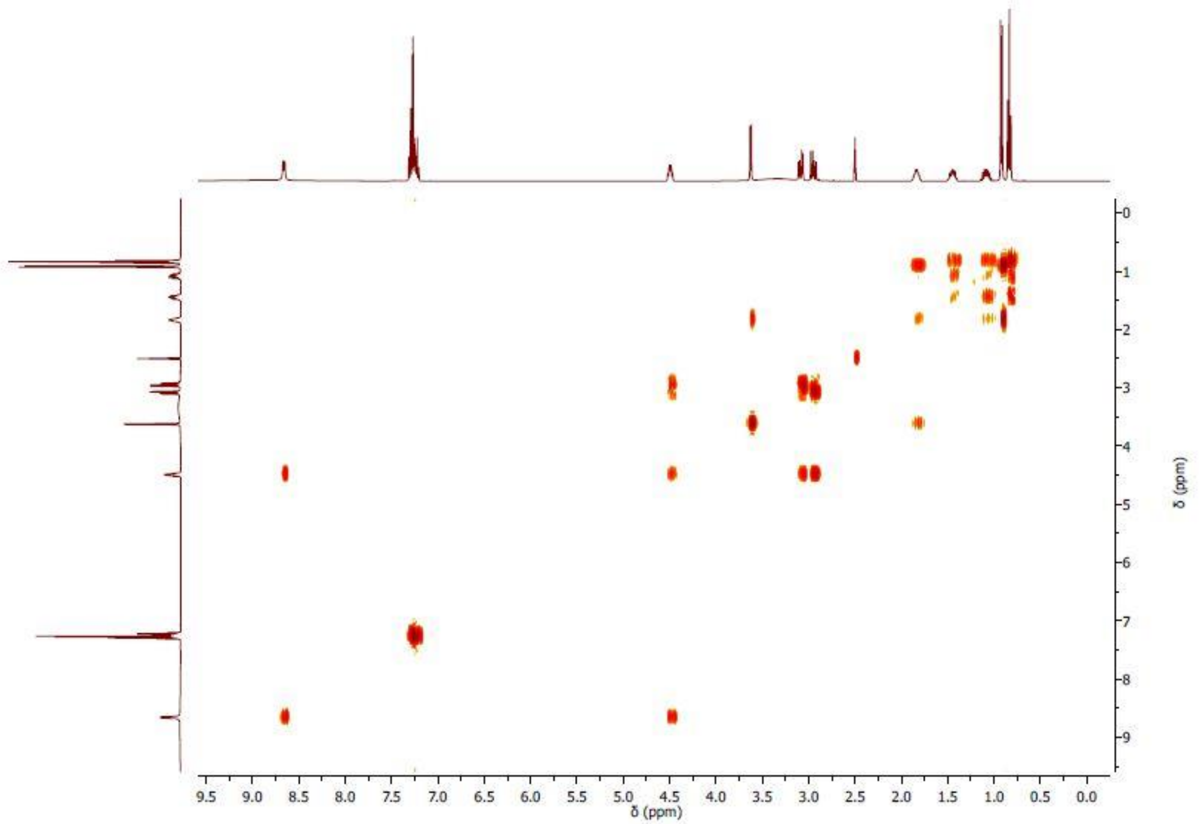

Fig. S2. gCOSY 2D-NMR spectrum of L-Ile-L-Phe.

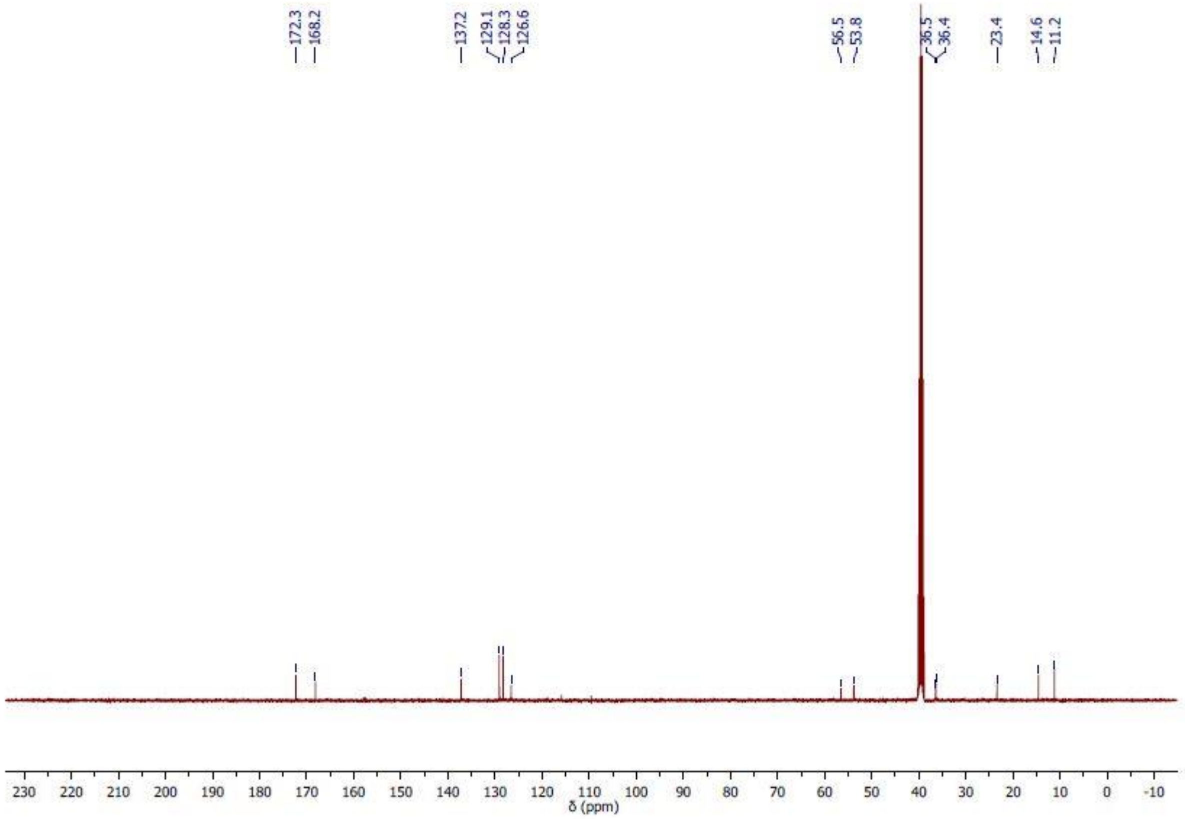

Fig. S3. ^{13}C-NMR spectrum of L-Ile-L-Phe.

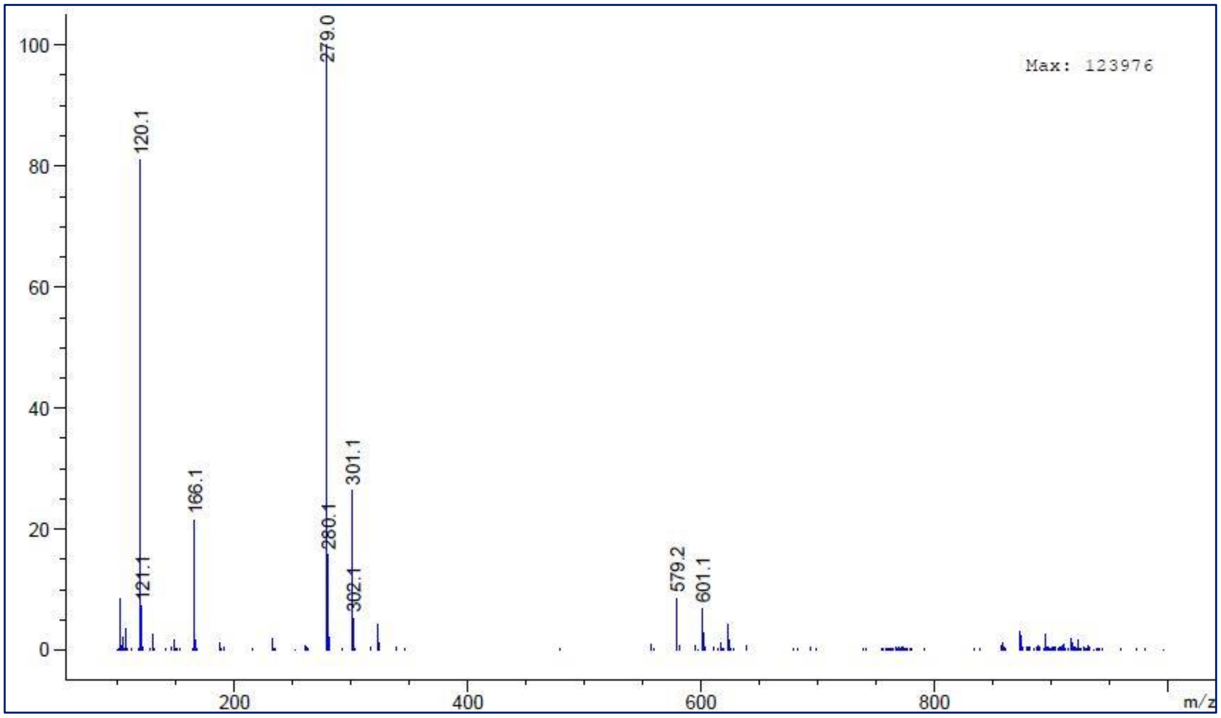

Fig. S4. ESI-MS spectrum of L-Ile-L-Phe (positive ion mode).

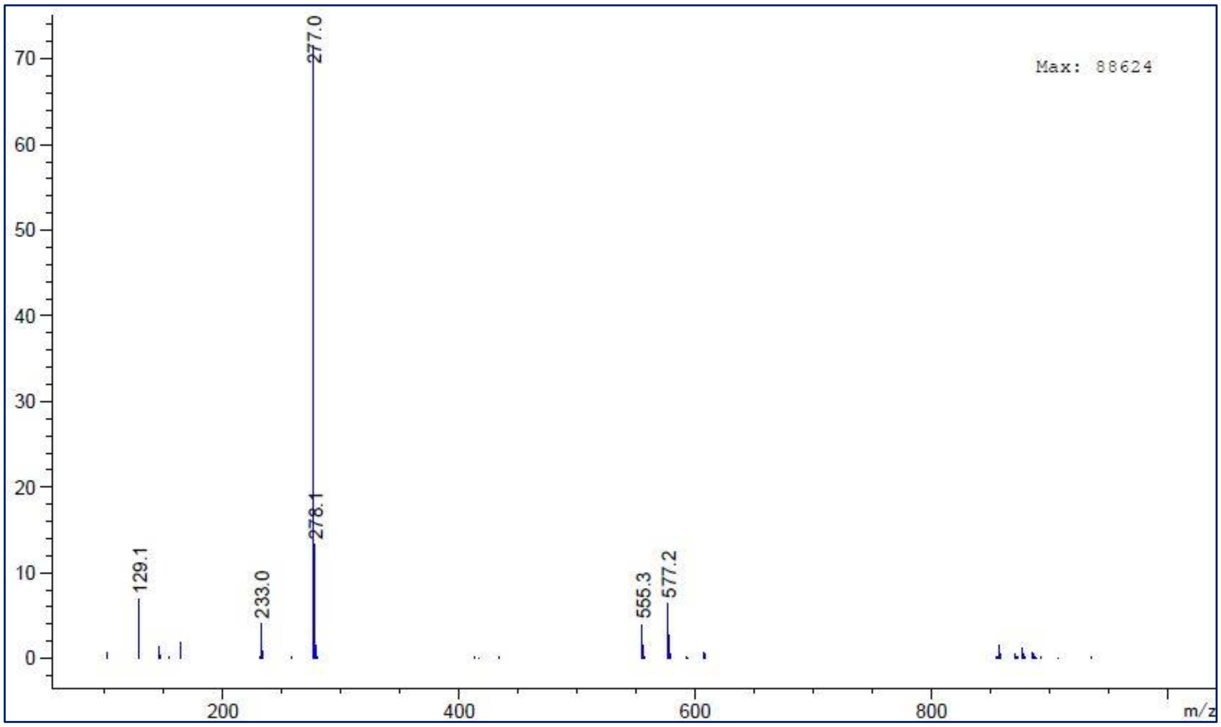

Fig. S5. ESI-MS spectrum of L-Ile-L-Phe (negative ion mode).

2. D-Ile-L-Phe spectroscopic data

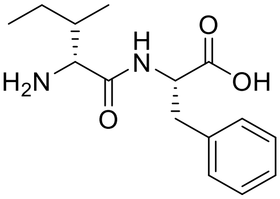

^{1}H NMR (400 MHz, DMSO-d_6, TMS), δ (ppm): 8.69 (d, *J* = 8.0 Hz, 1H, NH), 7.31 – 7.16 (m, 5H, Ar), 4.59 (ddd, *J* = 4.0, 8.0, 10.8 Hz, 1H, αCH Phe), 3.61 (d, *J* = 4.4 Hz, 1H, αCH Ile), 3.16 (dd, *J* = 14.0, 4.4 Hz, 1H, βCH_2), 2.83 (dd, *J* = 14.0, 10.8 Hz, 1H, βCH_2), 1.57 (m, 1H, βCH), 1.03 (ddddd, *J* = 3.2, 6.8, 6.8, 6.8, 13.6 Hz, 1H, γCH_2), 0.79 – 0.68 (m, 1H, γCH2), 0.64 (d, *J* = 6.4 Hz, 1H, γCH_3), 0.62 (dd, *J* = 6.8, 6.8 Hz, 1H, γCH_3). **^{13}C NMR** (100 MHz, DMSO-d_6, TMS), δ (ppm): 172.6, 167.8 (2 x CO); 137.3, 129.0, 128.2, 126.5, (Ar); 56.7, 53.6 (2 x αC); 36.9, 36.1 (2 x βC); 23.2, 14.3 (2 x ɣC); 11.3 (1 δC). **MS (ESI)**: m/z 279.1 $(M+H)^+$, 301.0 $(M+ Na)^+$; 277.1 $(M-H)^-$.

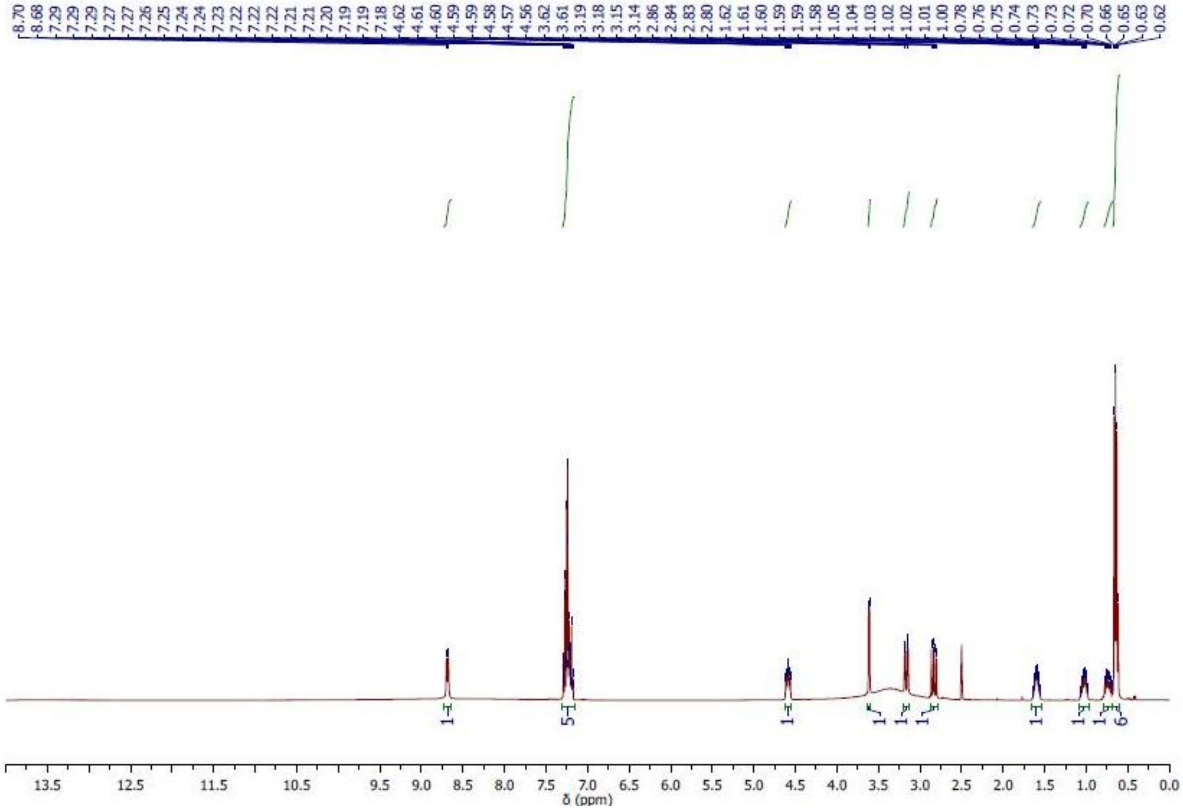

Fig. S6. ^{1}H-NMR spectrum of D-Ile-L-Phe.

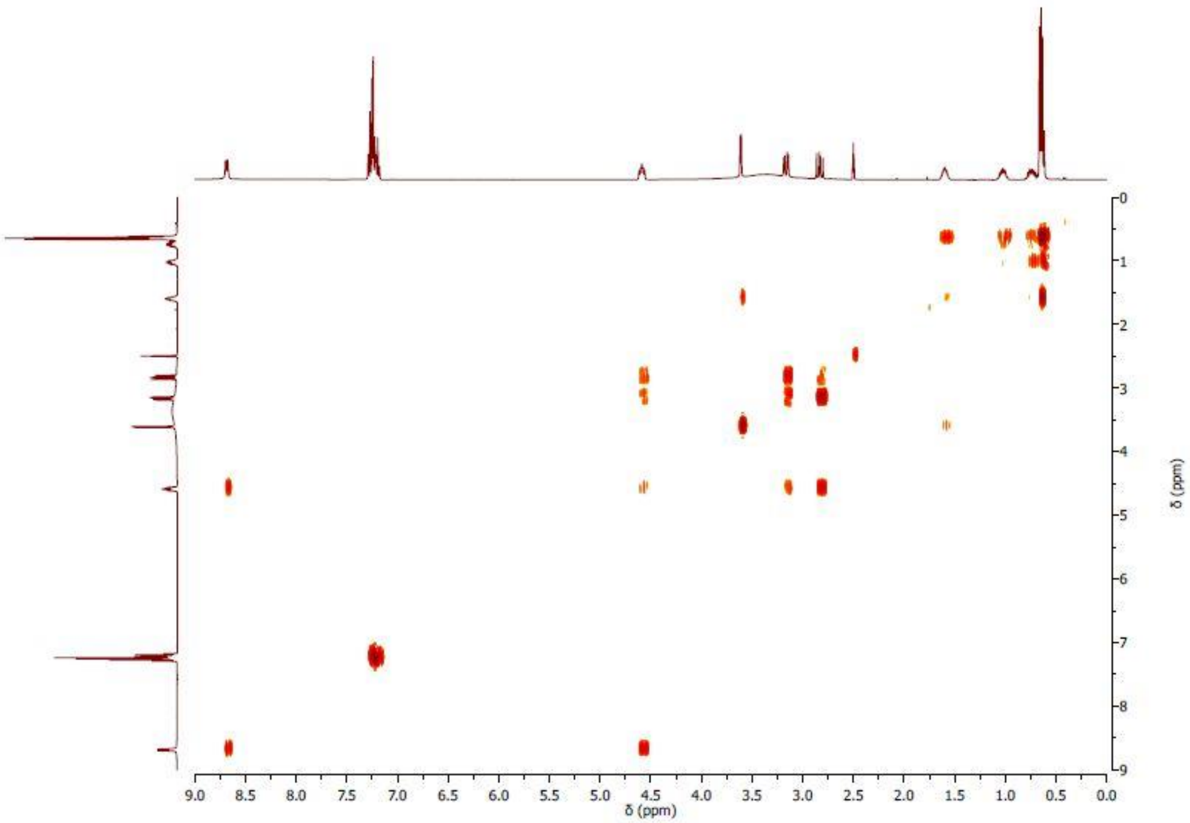

Fig. S7. gCOSY 2D-NMR spectrum of D-Ile-L-Phe.

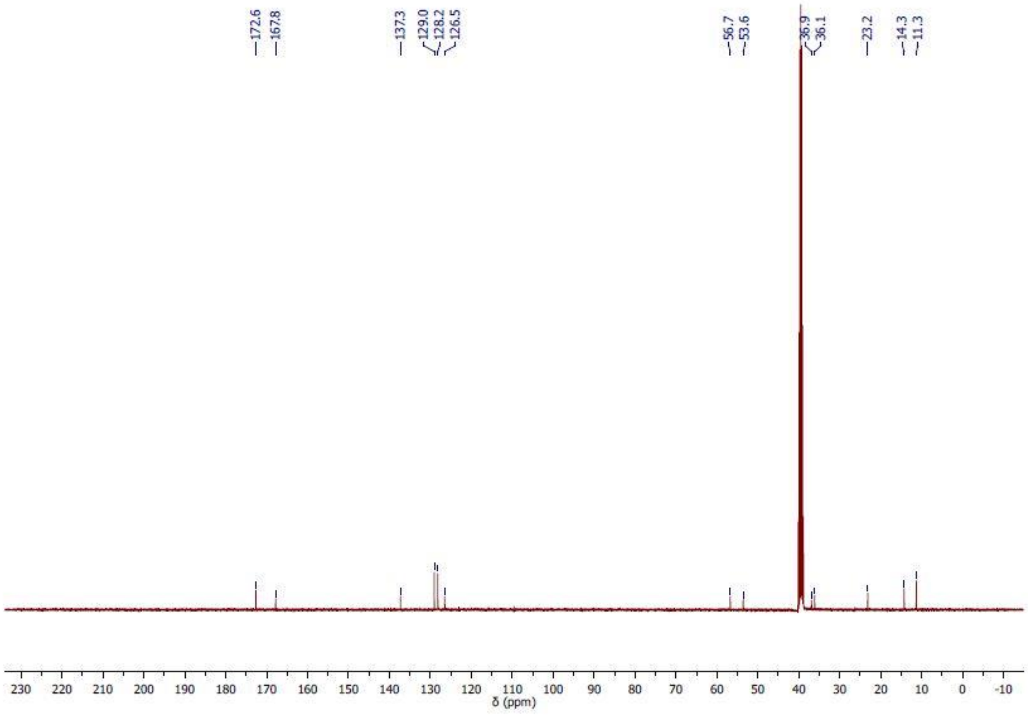

Fig. S8. ^{13}C-NMR spectrum of D-Ile-L-Phe.

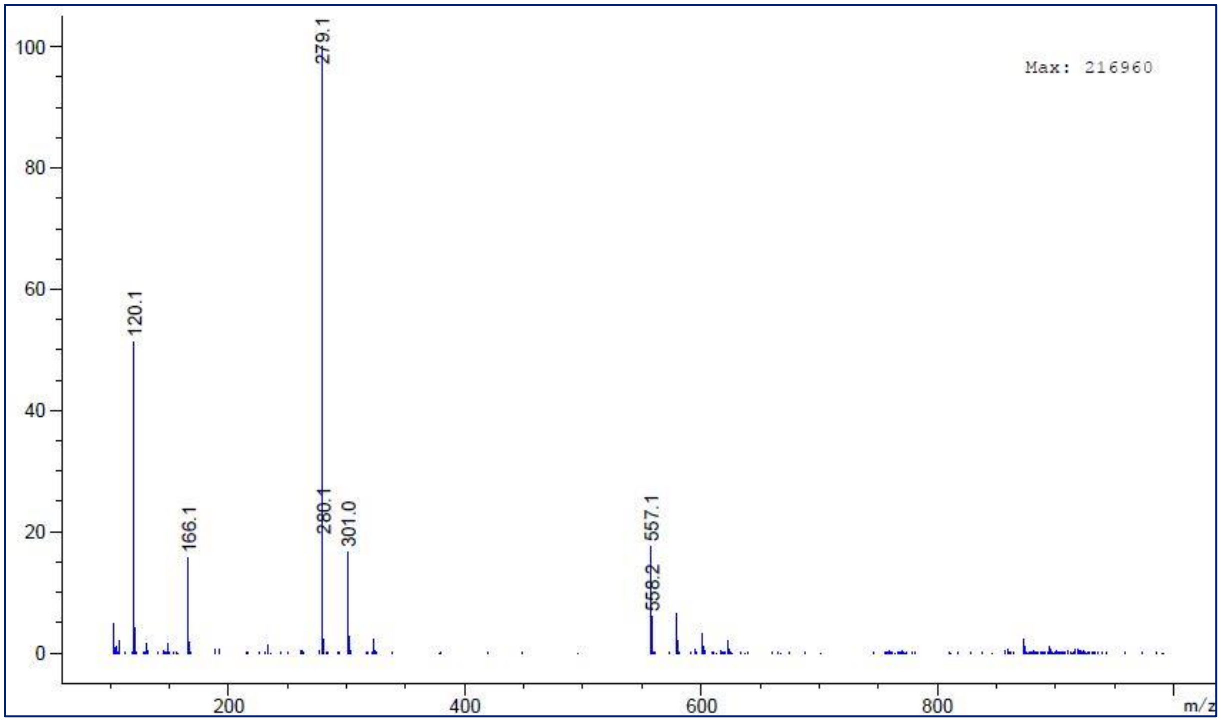

Fig. S9. ESI-MS spectrum of D-Ile-L-Phe (positive ion mode).

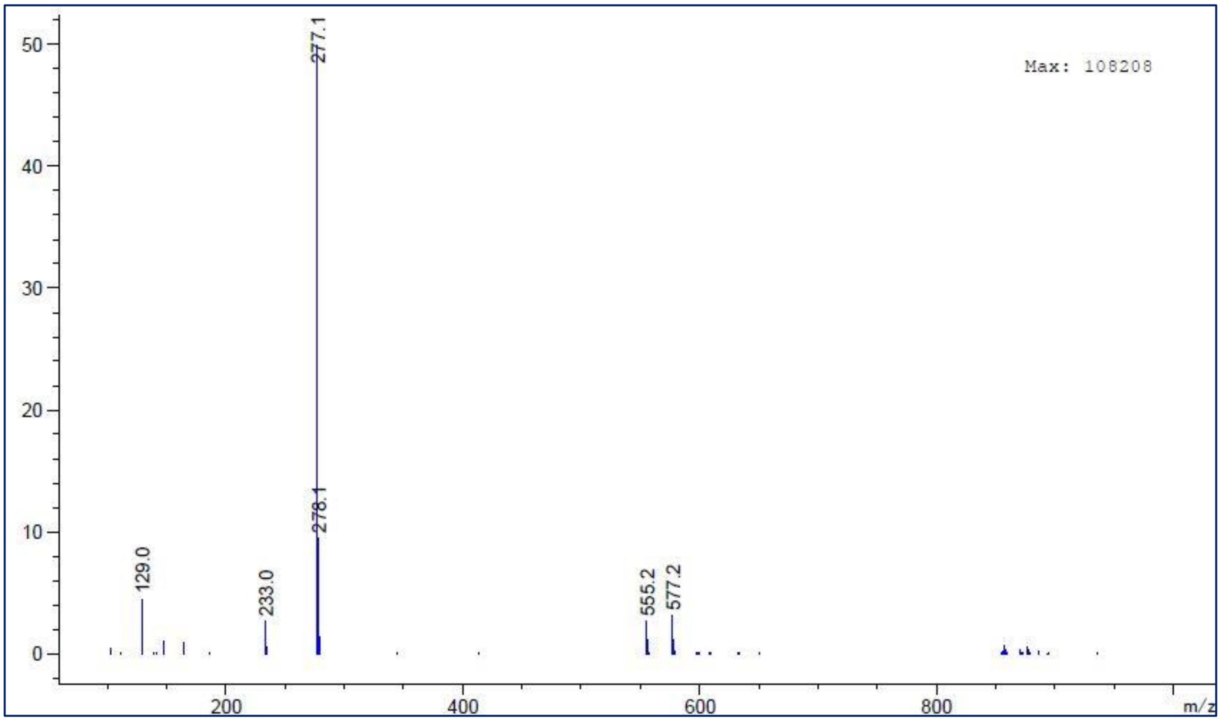

Fig. S10. ESI-MS spectrum of D-Ile-L-Phe (negative ion mode).

3. L-Phe-L-Ile spectroscopic data

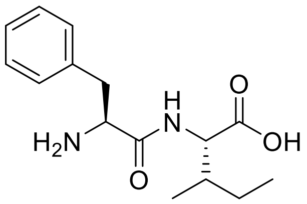

^{1}H NMR (400 MHz, DMSO-d_6, TMS), δ (ppm): 8.62 (d, J = 8.4 Hz, 1H, NH), 8.15 (s (br), 2H, NH_2), 7.36 – 7.24 (m, 5H, Ar), 4.23 (dd, J = 8.4, 5.6 Hz, 1H, αCH Ile), 4.13 (dd, J = 8.0, 5.6 Hz, 1H, αCH Phe), 3.09 (dd, J = 14.0, 8.0 Hz, 1H, $βCH_2$), 2.94 (dd, J = 14.0, 5.6 Hz, 1H, $βCH_2$), 1.81 (m, 1H, βCH), 1.44 (ddddd, J = 14.4, 7.2, 7.2, 7.2, 4.4 Hz, 1H, $γCH_2$), 1.21 (ddddd, J = 14.4, 7.2, 7.2, 7.2, 8.8 Hz, 1H, $γCH_2$), 0.89 (d, J = 6.8 Hz, 3H, $γCH_3$), 0.88 (dd, J = 7.2, 7.2 Hz, 3H, $δCH_3$). **^{13}C NMR** (100 MHz, DMSO-d_6, TMS), δ (ppm): 172.2, 168.1 (2 x CO); 134.7, 129.5, 128.5, 127.2 (Ar); 56.5, 53.1 (2 x αC); 37.0, 36.6 (2 x βC); 24.7, 15.5 (2 x ɣC); 11.4 (1 δC). **MS (ESI)**: m/z 279.1 $(M+H)^+$, 301.1 $(M+ Na)^+$; 277.1 $(M\text{-}H)^-$.

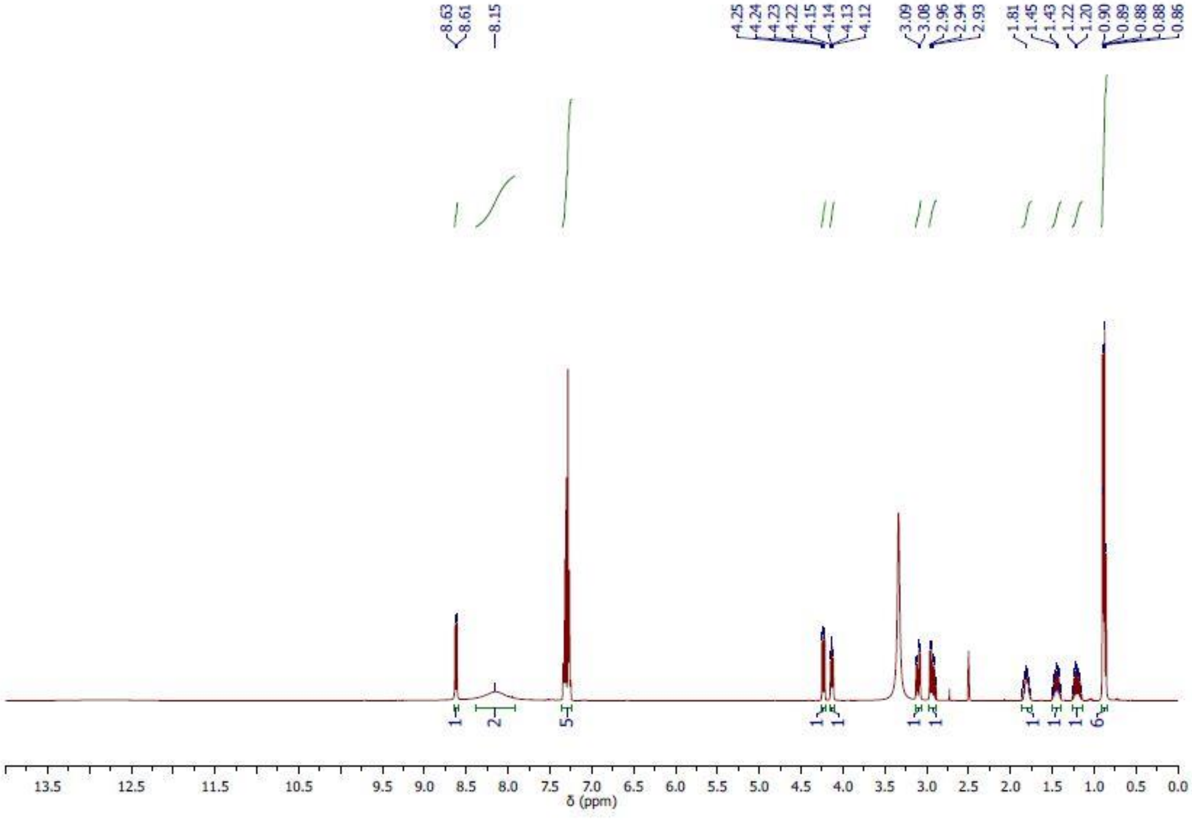

Fig. S11. ^{1}H-NMR spectrum of L-Phe-L-Ile.

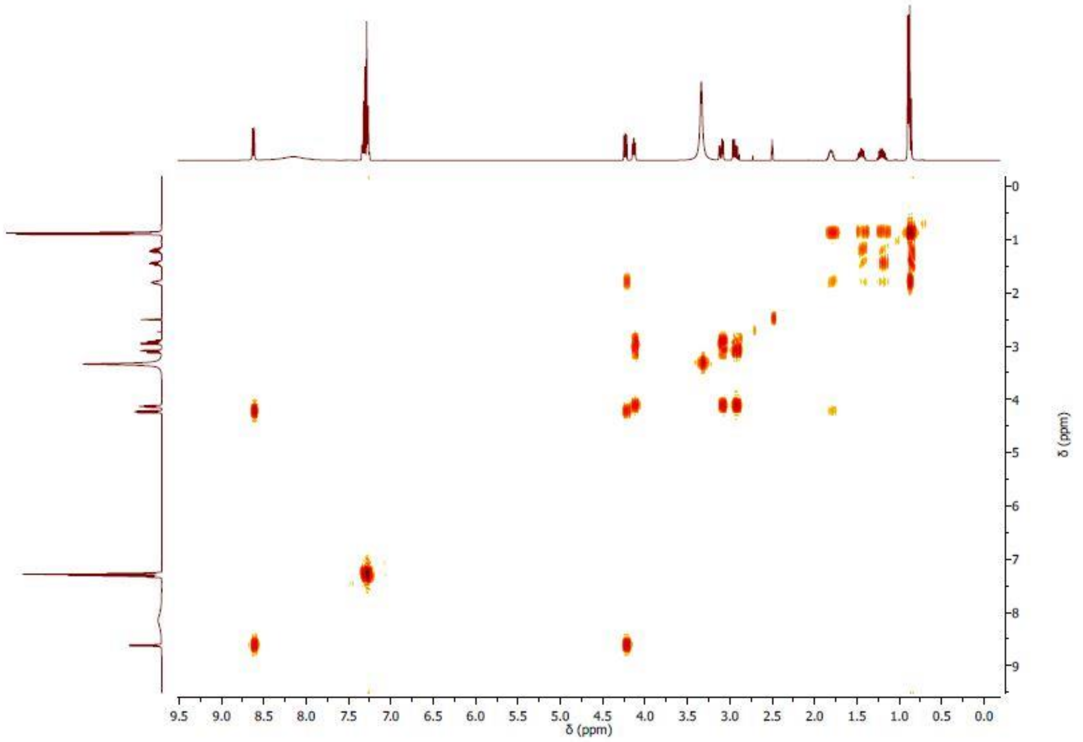

Fig. S12. gCOSY 2D-NMR spectrum of L-Phe-L-Ile.

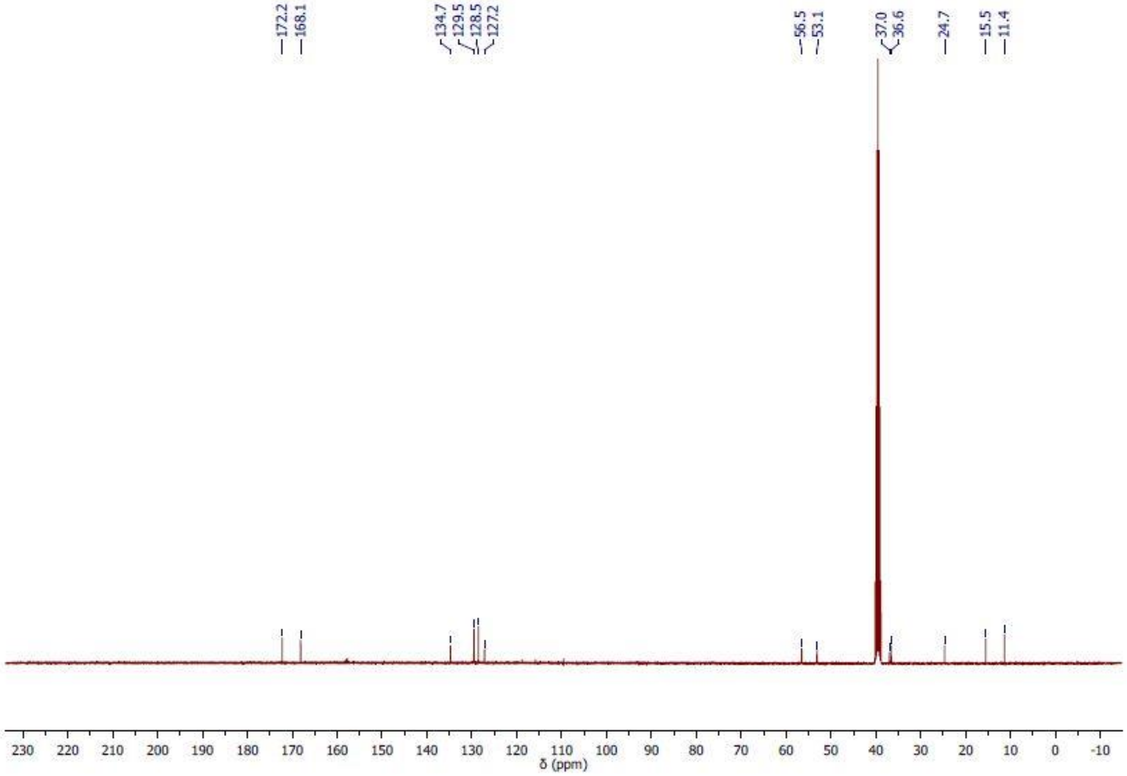

Fig. S13. ^{13}C-NMR spectrum of L-Phe-L-Ile.

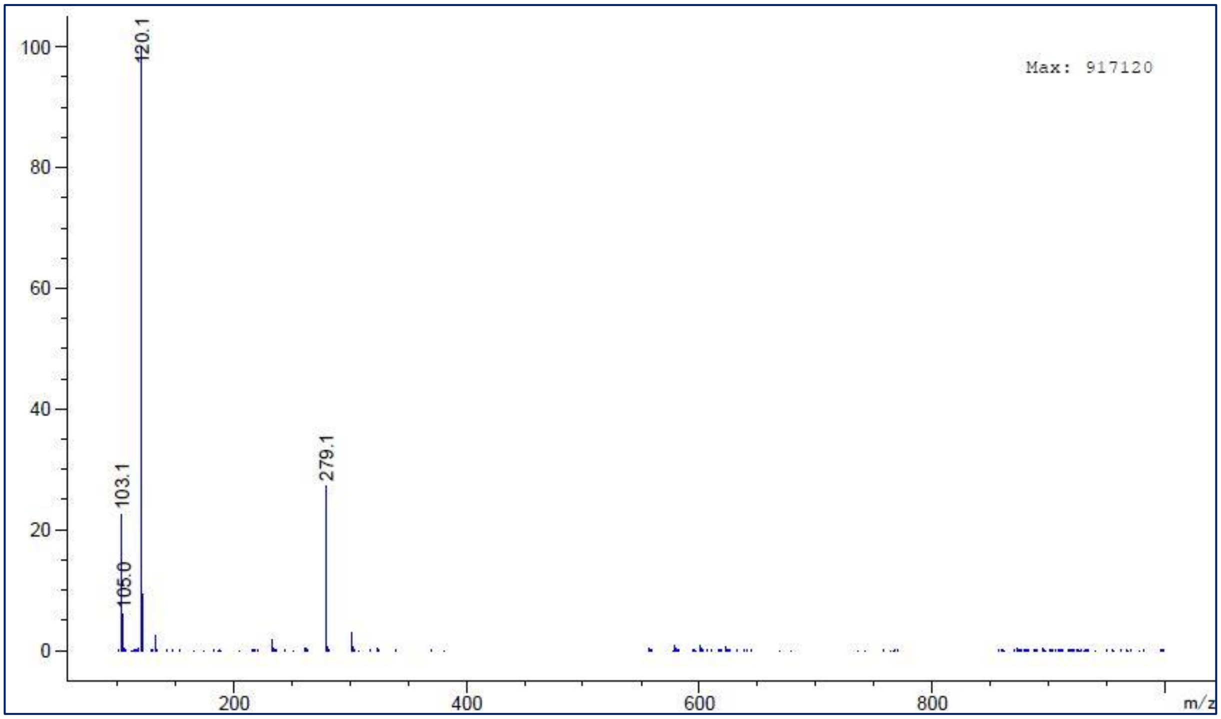

Fig. S14. ESI-MS spectrum of L-Phe-L-Ile (positive ion mode).

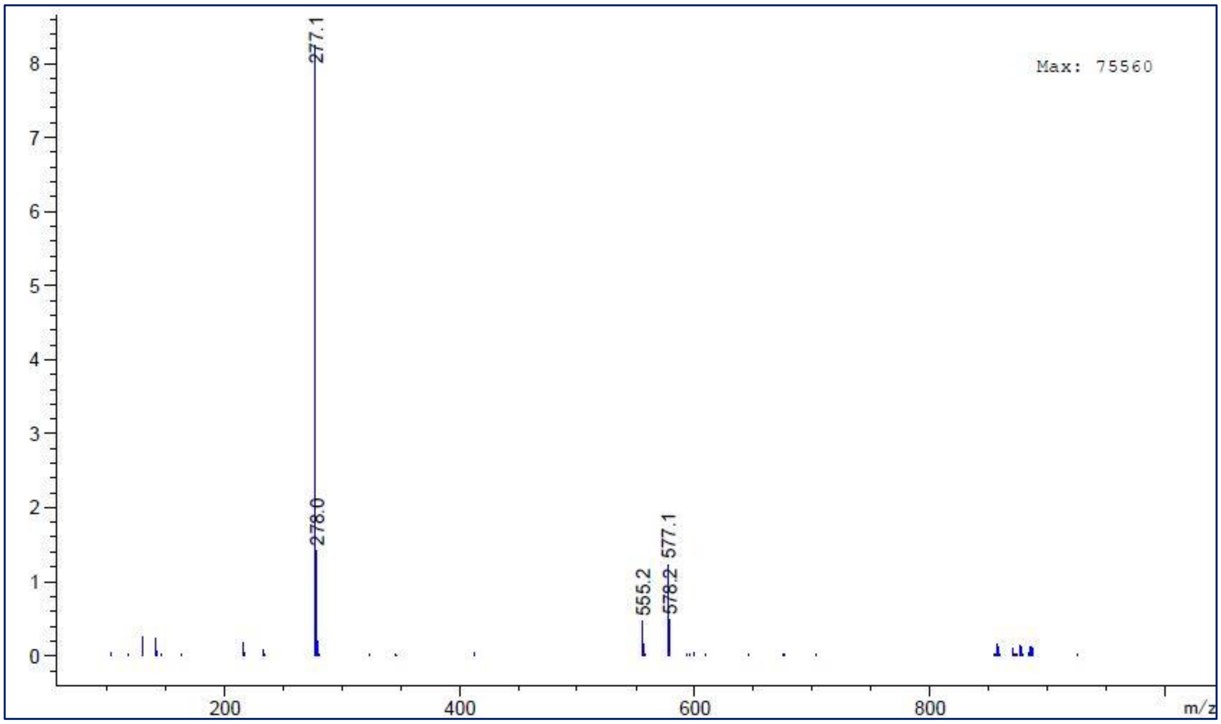

Fig. S15. ESI-MS spectrum of L-Phe-L-Ile (negative ion mode).

4. D-Phe-L-Ile spectroscopic data

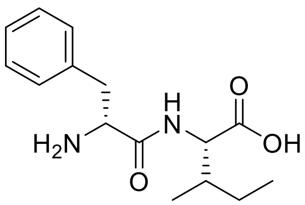

^{1}H NMR (400 MHz, DMSO-*d*$_6$, TMS), δ (ppm):): 8.58 (d, *J* = 8.4 Hz, 1H, NH), 7.36 – 7.22 (m, 5H, Ar), 4.18 (m, 2H, αCH Ile, αCH Phe), 3.03 (dd, *J* = 7.2, 13.6 Hz, 1H, βCH$_2$), 2.97 (dd, *J* = 7.6, 13.6 Hz, 1H, βCH$_2$), 1.61 (m, 1H, βCH), 1.22 (m, 1H, γCH$_2$), 0.97 (m, 1H, γCH$_2$), 0.77 (dd, *J* = 7.2, 7.2 Hz, 3H, CH$_3$), 0.69 (d, *J* = 6.8 Hz, 3H, CH$_3$). **^{13}C NMR** (100 MHz, DMSO-*d*$_6$, TMS), δ (ppm): 172.3, 168.0 (2 x CO); 134.8, 129.5, 128.5, 127.1 (Ar); 56.3, 53.1 (2 x αC); 37.5, 36.7 (2 x βC); 24.4, 15.3 (2 x ɣC); 11.2 (1 δC). **MS (ESI):** m/z 279.1 $(M+H)^{+}$, 301.1 $(M+ Na)^{+}$; 277.1 $(M-H)^{-}$.

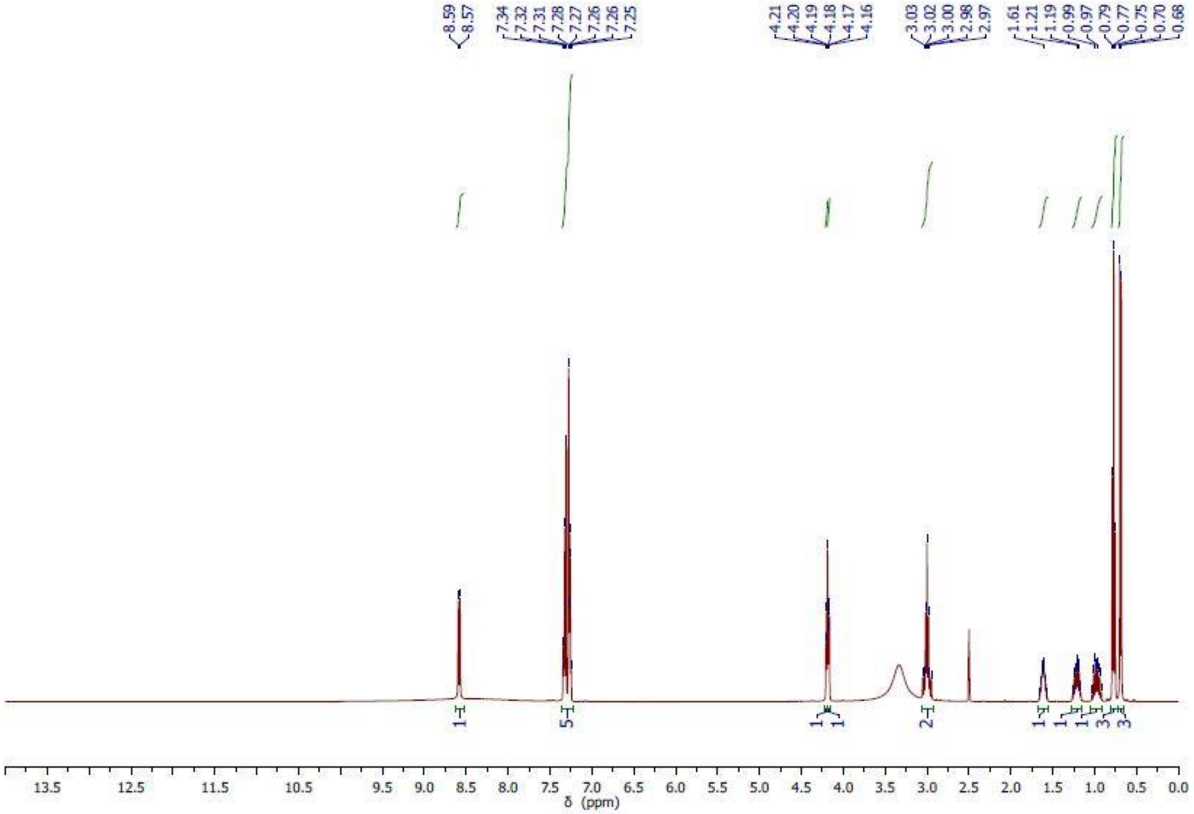

Fig. S16. ^{1}H-NMR spectrum of D-Phe-L-Ile.

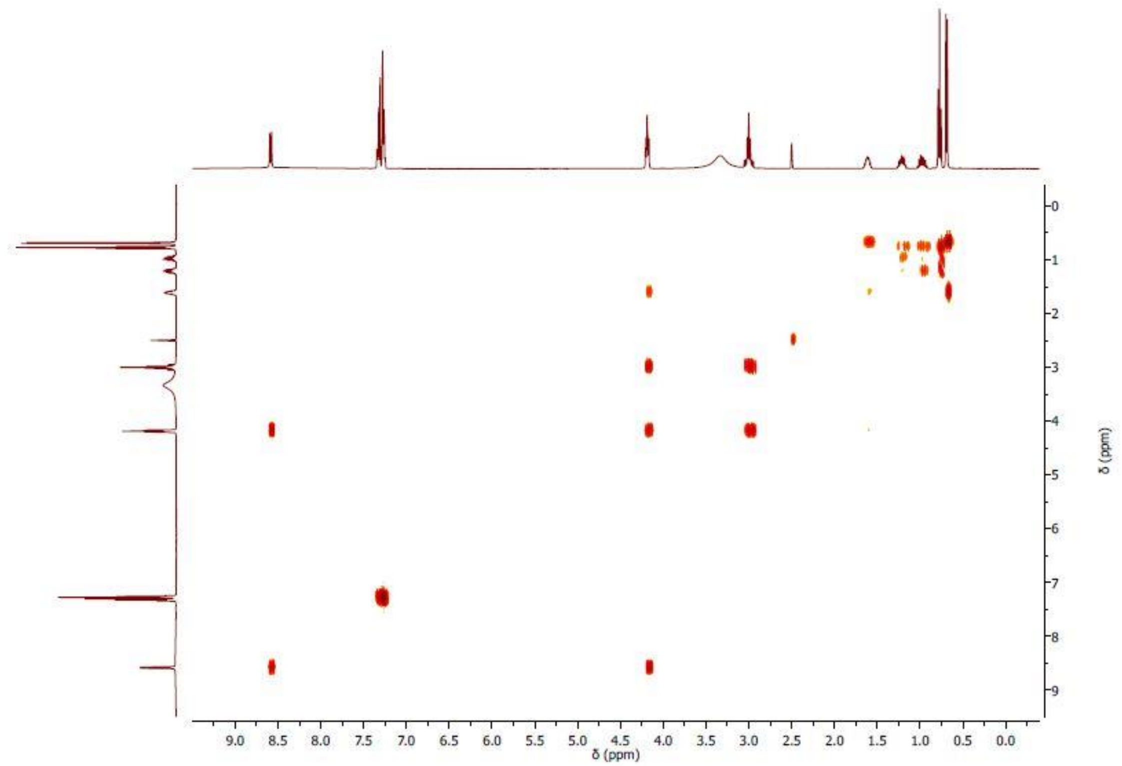

Fig. S17. gCOSY 2D-NMR spectrum of D-Phe-L-Ile.

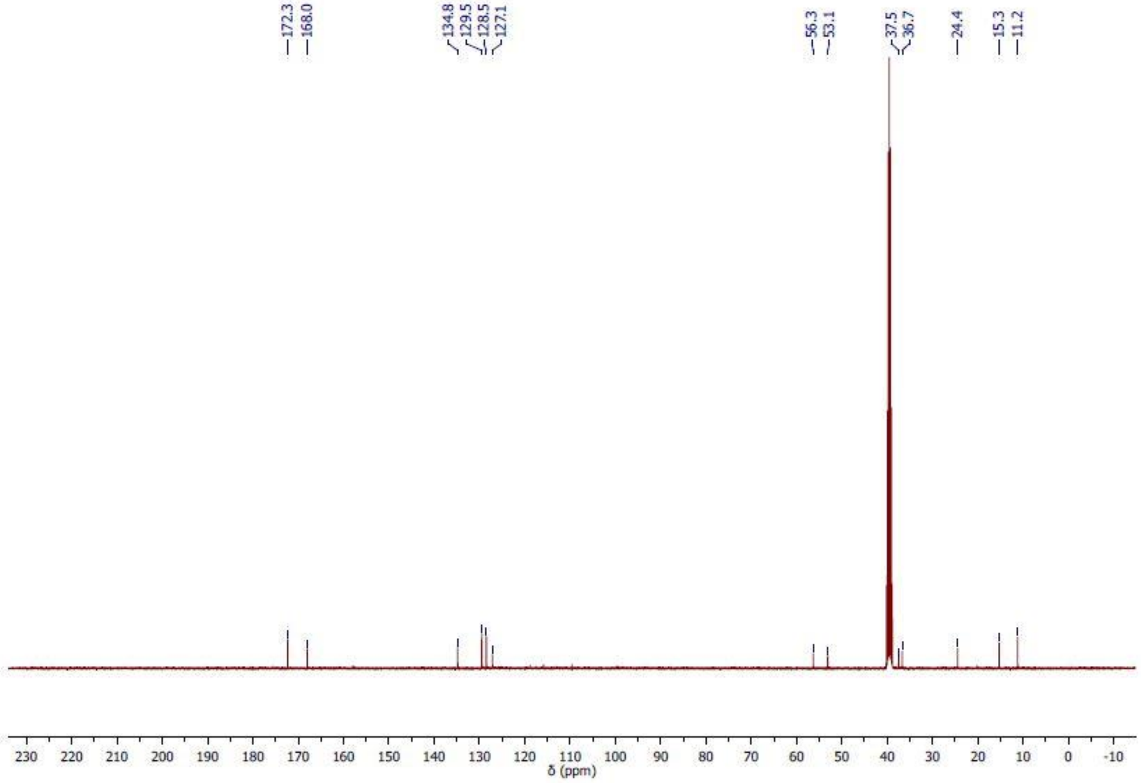

Fig. S18. ^{13}C-NMR spectrum of D-Phe-L-Ile.

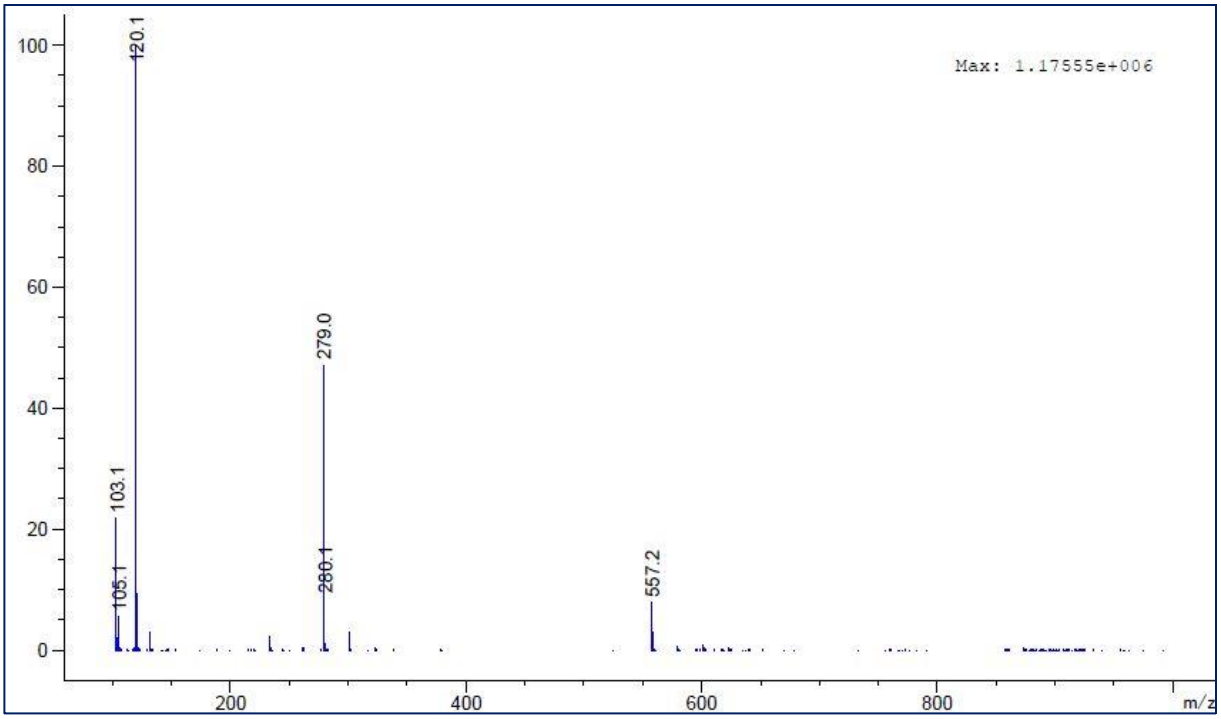

Fig. S19. ESI-MS spectrum of D-Phe-L-Ile (positive ion mode).

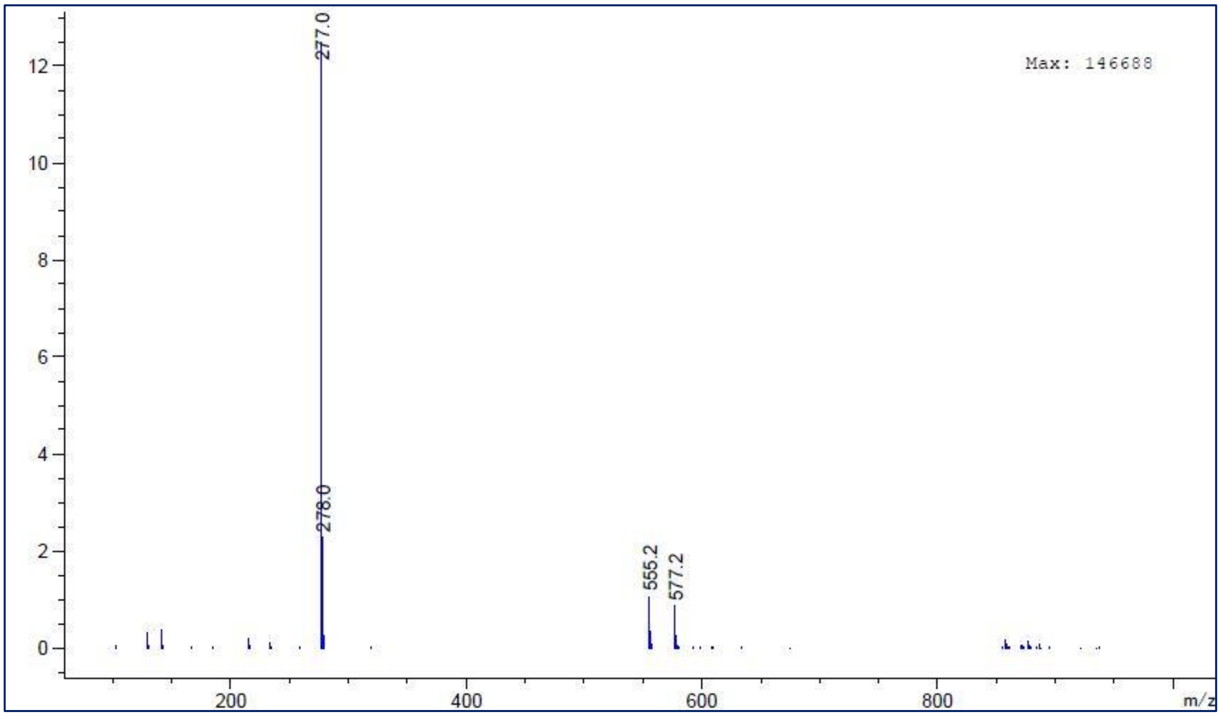

Fig. S20. ESI-MS spectrum of D-Phe-L-Ile (negative ion mode).

5. HPLC traces

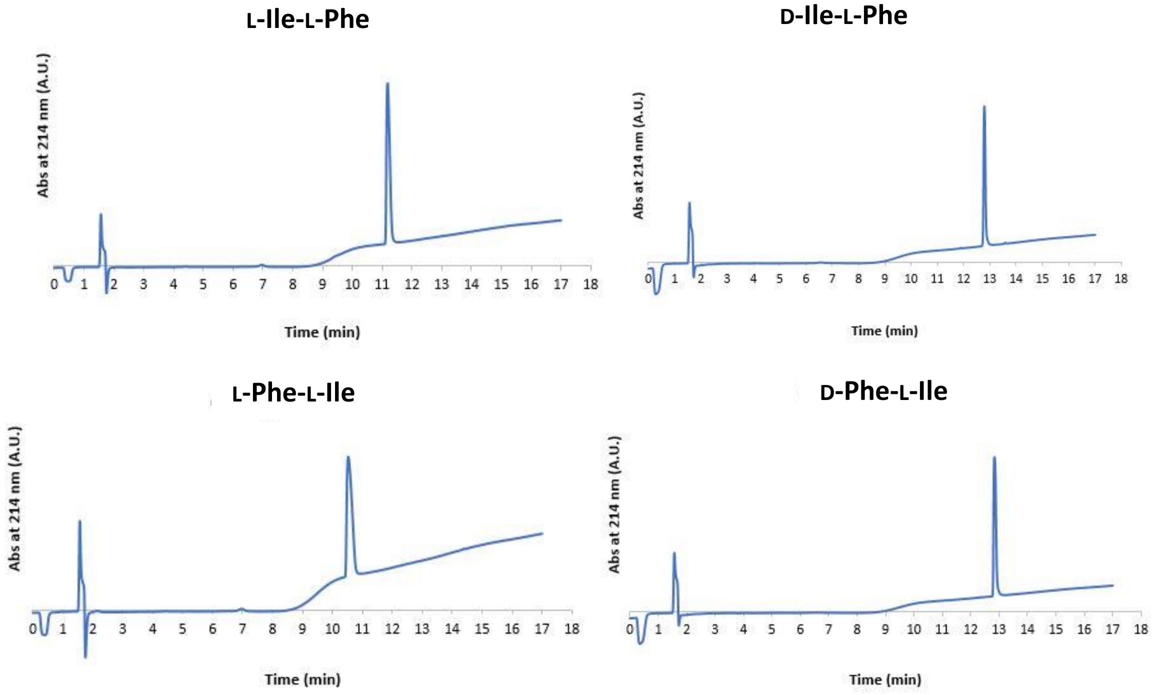

Fig. S21. HPLC traces for the four dipeptides (water/MeCN gradient system from 5% to 95% MeCN with 0.1% formic acid over 17 min, flow of 0.3 ml/min, C-18 column Luna, 5 µm, 100 Å, 150 x 2 mm, Phenomenex). Retention times are reported in the manuscript.

6. Rheology data for D-Phe-L-Ile hydrogel

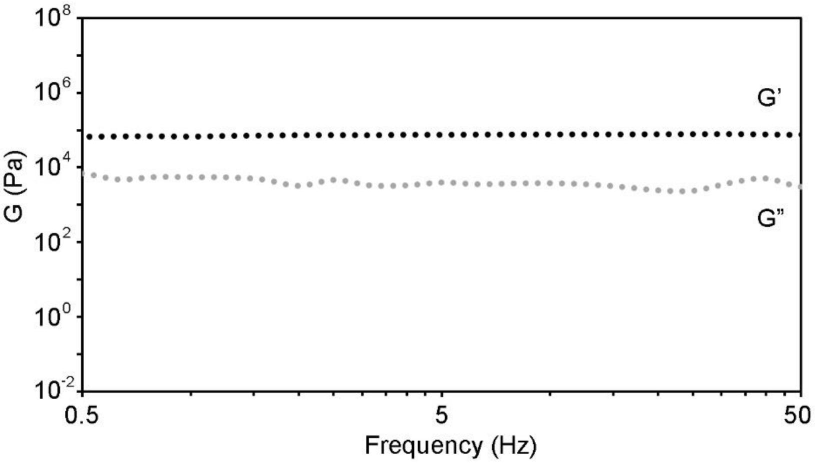

Fig. S22. Frequency sweep for D-Phe-L-Ile at 40 mM.

7. Rheology data for L-Ile-L-Phe (40 mM) viscous solution in PBS

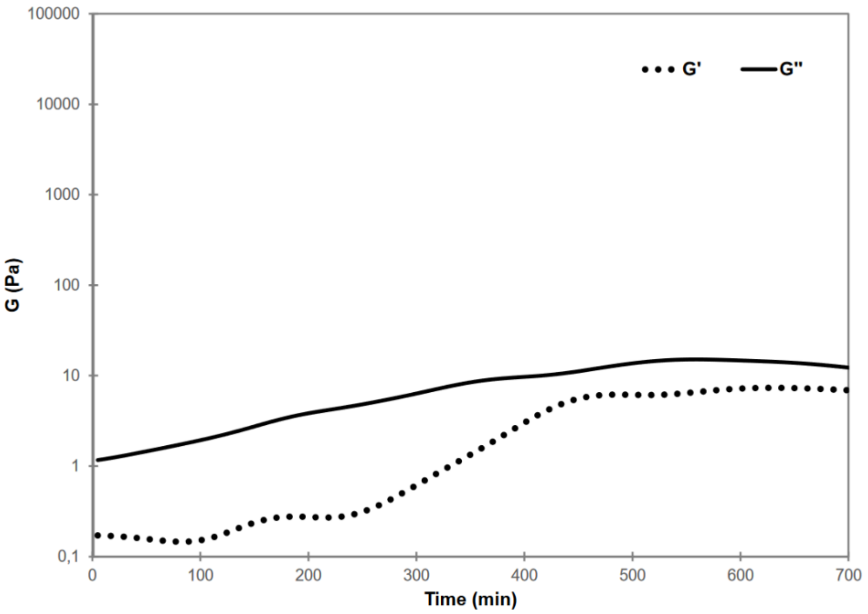

Fig. S23: Time sweep analysis of homochiral Ile-Phe (40 mM) viscous solution (G'' > G') in PBS.

8. Single-crystal XRD data

D-Ile-L-Phe – CCDC109139

Description. The asymmetric unit contains a single molecule of the peptide in its zwitterion form and 3 molecules of water, one of which in a 2-fold symmetry axis present in the unit cell. A total of 4 molecules of peptide, related by symmetry operators of the *C2* space group, are present in the unit cell (Fig. S24). The crystal packing shows a separation between regions with prevalence of hydrophobic groups, *i.e.* the phenyl moiety and the Ile side chain, and regions with hydrophilic interactions involving the backbone of the peptides and the water molecule (Fig. S25). Hydrophilic interactions between the peptides are mediated by water molecules both along the *b* crystallographic direction and along the *a* crystallographic direction. In the *b* direction, a water molecule forms two hydrogen bonds with the carbonyl and the amide moieties of two different peptides (Fig. S26a). Along the *a* direction, peptides are connected through salt bridges between the N-terminus and the C-terminus (Fig. S26b). In addition, further interactions involving water molecules saturate the hydrogen bonding potential of the peptide (Fig. S26b). Only weak hydrophobic interactions are present between the phenyl moieties and the Ile side chains.

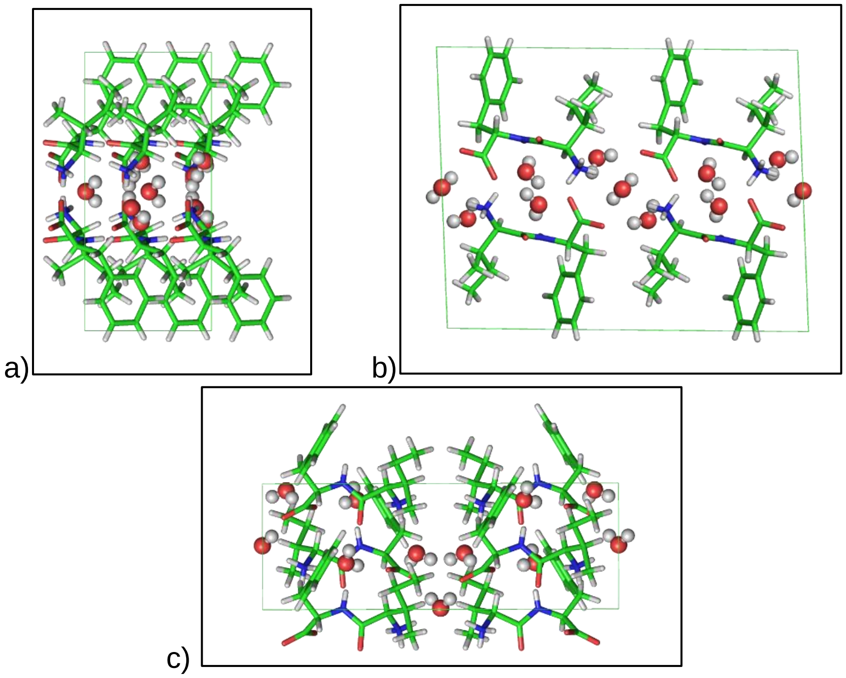

Fig. S24. Unit cell of crystals of D-Ile-L-Phe. Crystal packing of the dipeptide. Views along the *a* crystallographic axis (a), the *b* crystallographic axis (b) and the *c* crystallographic axis (c). Peptide molecules are shown as sticks, water molecules are shown as spheres. Carbon atoms are shown in green, oxygen atoms in red, nitrogen atoms in blue, hydrogen atoms in white.

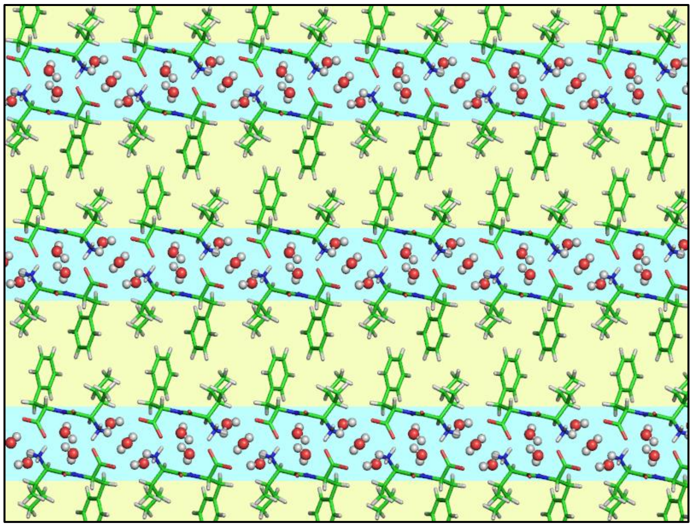

Fig. S25: Crystal packing with alternating hydrophilic and hydrophobic regions. Hydrophobic layers are shown with a yellow background, hydrophilic layers with a blue background. Crystal packing is shown in the plane perpendicular to the *b* crystallographic direction.

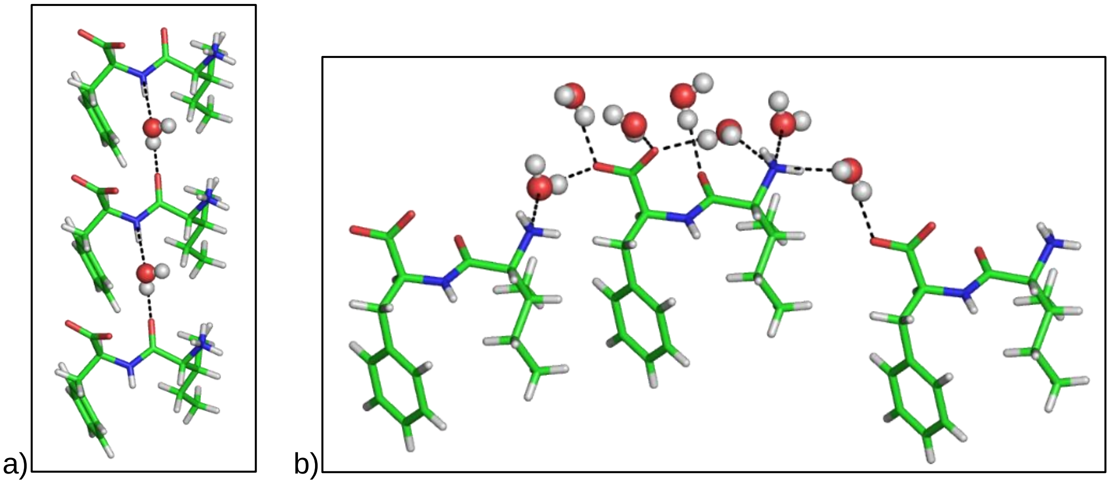

Fig. S26: Hydrophilic interactions. Hydrogen bonding interactions (a) along the *b* crystallographic direction and (b) along the *a* crystallographic direction are marked with black dashes.

<u>Crystallographic details</u>. A stick-shaped single crystal of the peptide was collected with a loop, cryoprotected by dipping the crystal in glycerol, and stored frozen in liquid nitrogen. The crystal was mounted on the diffractometer at the synchrotron Elettra, Trieste (Italy), beamline XRD1, using the robot present at the facility. Temperature was kept at 100 K by a stream of nitrogen on the crystal. Diffraction data were collected by the rotating crystal method using synchrotron radiation, wavelength 0.70 Å, rotation interval 1°/image, crystal-to-detector distance of 85 mm. A total of 360 images were collected. Reflections were indexed and integrated using the XDS package [1], space group *C2* was determined using POINTLESS [2] and the resulting data set was scaled using AIMLESS [3]. Phase information were obtained by direct methods using the software SHELXT [4]. Refinements cycles were conducted with SHELXL-14 [4], operating through the WinGX GUI [5], by full-matrix least-squares methods on F^2. Unit cell parameters and scaling statistics are reported in Table S1. The asymmetric unit contains a molecule of the peptide and 3 molecules of water, one of which located in a 2-fold symmetry axis present in the unit cell. Hydrogen atoms of the peptide were added at geometrically calculated positions and refined isotropically, with thermal parameters dependent on those of the attached atom. Hydrogen atoms of the water molecules were added taking into account the residual electron density and the geometry of interactions with the peptide molecules. All the atoms, except the hydrogen atoms, within the asymmetric unit have been refined with anisotropic thermal parameters. During refinement, no restraints were applied on distances, angles or thermal parameters of non-hydrogen atoms. Restraints on on bond distances and angles of the water molecules were added using the DFIX and DANG cards in SHELXL-14 [5]. Refinement statistics are reported in Table S1.

D-Phe-L-Ile – CCDC109138

<u>Description</u>. The asymmetric unit contains two molecules of the peptide in its zwitterion form and 4 molecules of water. A total of 18 molecules of peptide, related by symmetry operators of the *R3* space group, are present in the unit cell (Fig. S27). The crystal packing shows a clear separation between hydrophilic and hydrophobic regions

(Fig. S28). In particular, peptides arrange in a tubular fashion along the *c* crystallographic direction, through hydrophilic interactions (Fig. S29). The tube-like superstructures are hollow and contain solvent only partially ordered, and therefore partially visible in the crystal structure. Molecules forming the walls of the tube are related by the 3-fold crystallographic axis, a symmetry element belonging to the *R3* space group of the crystal, but also by a 6-fold non-crystallographic symmetry that relates the two independent peptide molecules present in the unit cell (Fig. S30) and two of the independent water molecules with the other two. Tubes held together by hydrophilic interactions interact with each other through weaker hydrophobic interactions of the phenyl and 2-methylpropyl side chains. In particular, intermolecular (black dashes in Fig. S31) and intramolecular (magenta dashes in Fig. S31) CH-π interactions are present between molecules related by a 3_1 crystallographic axis, with distances between the hydrogen atom and the center of the phenyl group of about 3 Å.

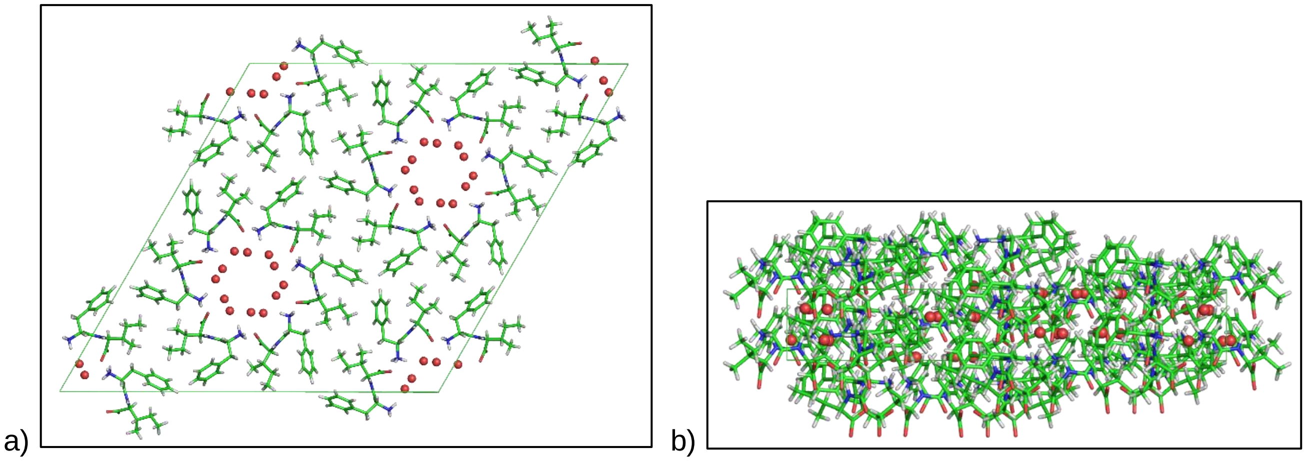

Fig. S27: Unit cell of crystals of the dipeptide. Views along the *a* crystallographic axis (a) and the *c* crystallographic axis (b). Peptide molecules are shown as sticks, water molecules are shown as spheres.

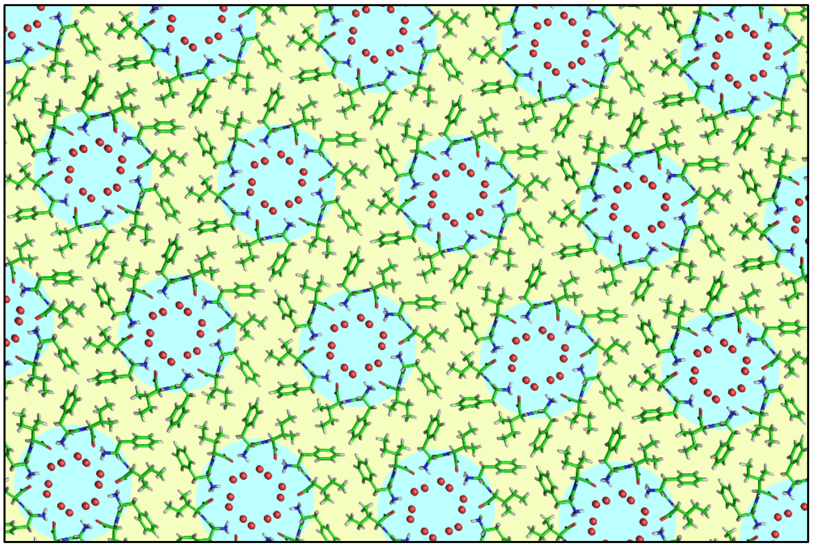

Fig. S28: Crystal packing. Peptide molecules form tubes along the *c* crystallographic direction through hydrophilic interactions, in blue. The side chains of the residues of each tube face those of the neighboring ones, interacting through hydrophobic contacts, in yellow.

a) b)

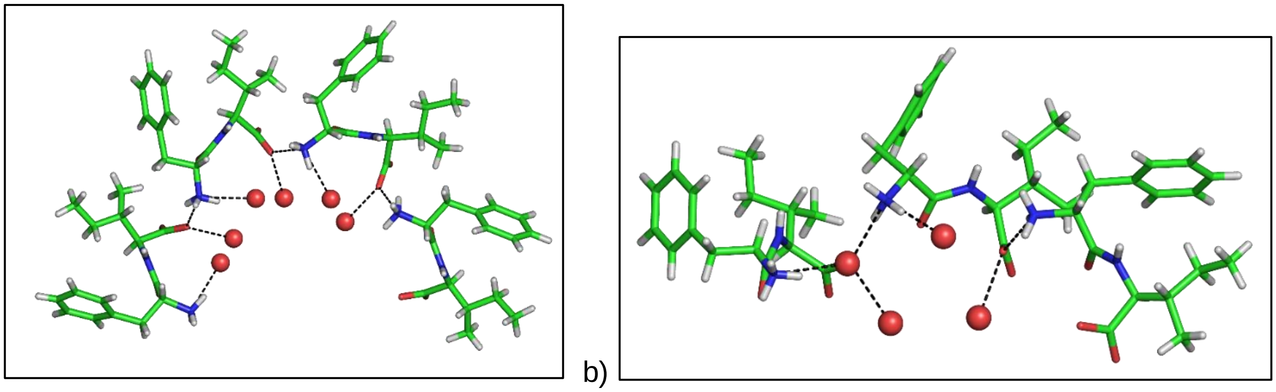

Fig. S29: Hydrophilic interactions between peptide molecules and water molecules in the crystal structure. Views of the hydrophilic interactions present in the crystal structure along the *c* crystallographic axis (a) and in the perpendicular direction (b). Salt bridge interactions connect the N- and C- termini of peptide molecules, while the water molecules are connected with the peptides through hydrogen bonds.

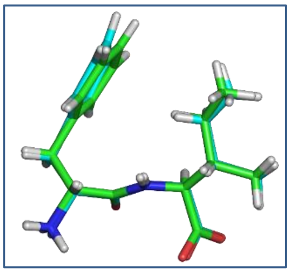

Fig. S30: Superimposition of the two crystallographically independent peptide molecules present in the crystal structure. The conformation of both the main chain and the side chain of the crystallographically independent peptide molecules present in the asymmetric unit of the crystal structure is similar. In addition, the two molecules are related by a 6-fold non-crystallographic rotation axis.

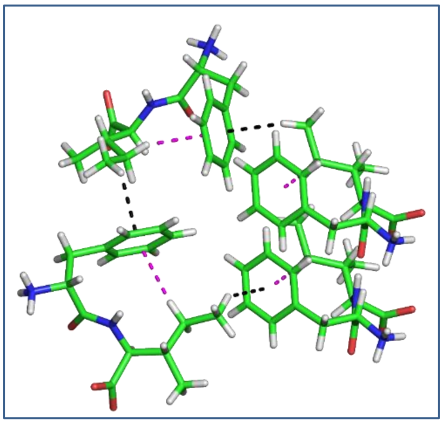

Fig. S31: Hydrophobic interactions between the peptide molecules present in the crystal structure. Phenyl and 1-methylpropyl groups form intermolecular (black dashes) and intramolecular (magenta dashes) CH-π interactions.

Crystallographic details. A stick-shaped single crystal of the peptide was collected with a loop, cryoprotected by dipping the crystal in glycerol and stored frozen in liquid nitrogen. The crystal was mounted on the diffractometer at the synchrotron Elettra, Trieste (Italy), beamline XRD1, using the robot present at the facility. Temperature was kept at 100 K by a stream of nitrogen on the crystal. Diffraction data were collected by the rotating crystal method using synchrotron radiation, wavelength 0.70 Å, rotation interval 1°/image, crystal-to-detector distance of 85 mm. A total of 175 images were collected. Reflections were indexed and integrated using the XDS package [1], space group *R3* was determined using POINTLESS [2] and the resulting data set was scaled using AIMLESS [3]. Phase information were obtained by direct methods using the software SHELXT [4]. Refinements cycles were conducted with SHELXL-14 [4], operating through the WinGX GUI [5], by full-matrix least-squares methods on F^2. Unit cell parameters and scaling statistics are reported in Table S1. The asymmetric unit contains 2 molecules of the peptide and 4 water molecules. All the atoms within the asymmetric unit, except the hydrogen atoms, have been refined with anisotropic thermal parameters. Hydrogen atoms of the peptide molecules were added at geometrically calculated positions and refined isotropically, with thermal parameters dependent on those of the attached atom. Hydrogen atoms of the water molecules could not be located. Disordered solvent molecules present in the large cavities of the structure were too difficult to be modelled, but their contribution was taken into account using the SQUEEZE/PLATON procedure [6]. Residual electron densities corresponding to 106 electrons/cell were found in the voids of the crystal, corresponding to 4% of the cell volume. Refinements using reflections modified by the SQUEEZE procedure behaved well and R-factors were reduced from 11% to 10%. Refinement statistics are reported in Table S1.

Table S1: Crystallographic data for CCDC10939 and CCDC10938.

	D-Ile-L-Phe CCDC109139	D-Phe-L-Ile CCDC109138
Formula	$C_{15}H_{22}N_2O_3 \cdot 2.5H_2O$	$C_{15}H_{22}N_2O_3 \cdot 2H_2O$ [+solvent]
Temperature (K)	100	100
Wavelength (Å)	0.7	0.7
Crystal system	Monoclinic	Trigonal
Space group	*C 2*	*R 3*
a (Å)	18.779(4)	40.002(6)
b (Å)	6.641(1)	40.002(6)
c (Å)	14.634(3)	5.6070(11)
α (°)	90	90
β (°)	92.74(3)	90
γ (°)	90	120
V ($Å^3$)	1822.9(6)	7770(3)
Z, ρcalc (g/cm^3)	4, 1.178	18, 1.209
μ (mm^{-1})	0.085	0.087
F (000)	700	3060
Data collection θ range	2.486 - 29.82	1.737 - 24.835
Refl. Collected / unique	15804 / 5129	22519 / 9324
Rint	0.080	0.0801
Completeness (%)	98.1	94.8
Data/Restraints/Parameters	5129 / 9 / 228	9324 / 1 / 400
GooF	1.027	1.019
R1, wR2 [$I>2\sigma(I)$]	0.0549 / 0.1438	0.097 / 0.2586
R1, wR2 all data	0.055 / 0.144	0.1365 / 0.2959

References:

[1] Kabsch, W. Acta Crystallogr., Sect. D. 2010, 66, 125−132.
[2] Evans, P. R. Acta Crystallogr., Sect. D. 2006, 62, 72−82.
[3] Evans, P. R., Murshudov, G. N. Acta Crystallogr., Sect. D. 2013, 69, 1204−14.
[4] Sheldrick, G. M. Acta Crystallogr., Sect. C. 2015, 71, 3–8.
[5] Farrugia, L.J. J. Appl. Cryst. 2012, 45, 849−854.
[6] Spek, A.L. ActaCrystallogr., Sect. C. 2015, 71, 9-18.

Table S2: Dipeptide dihedral angles form the crystal-structure data.

	ψ(1): N(1)Cα(1)C(1)N(2)	ω(1-2): Cα(1)C(1)N(2)Cα(2)	ϕ(2): C(1)N(2)Cα(2)C(2)
L-Ile-L-Phe	149.92	170.64	49.49
D-Ile-L-Phe	-139.20	179.36	-68.58
L-Phe-L-Ile	114.92	-171.45	52.31
	164.36	-178.51	-83.13
D-Phe-L-Ile	-130.51	-178.01	-69.68
	-132.11	-178.11	-69.74

9. High-resolution transmission electron microscopy (HR-TEM) data

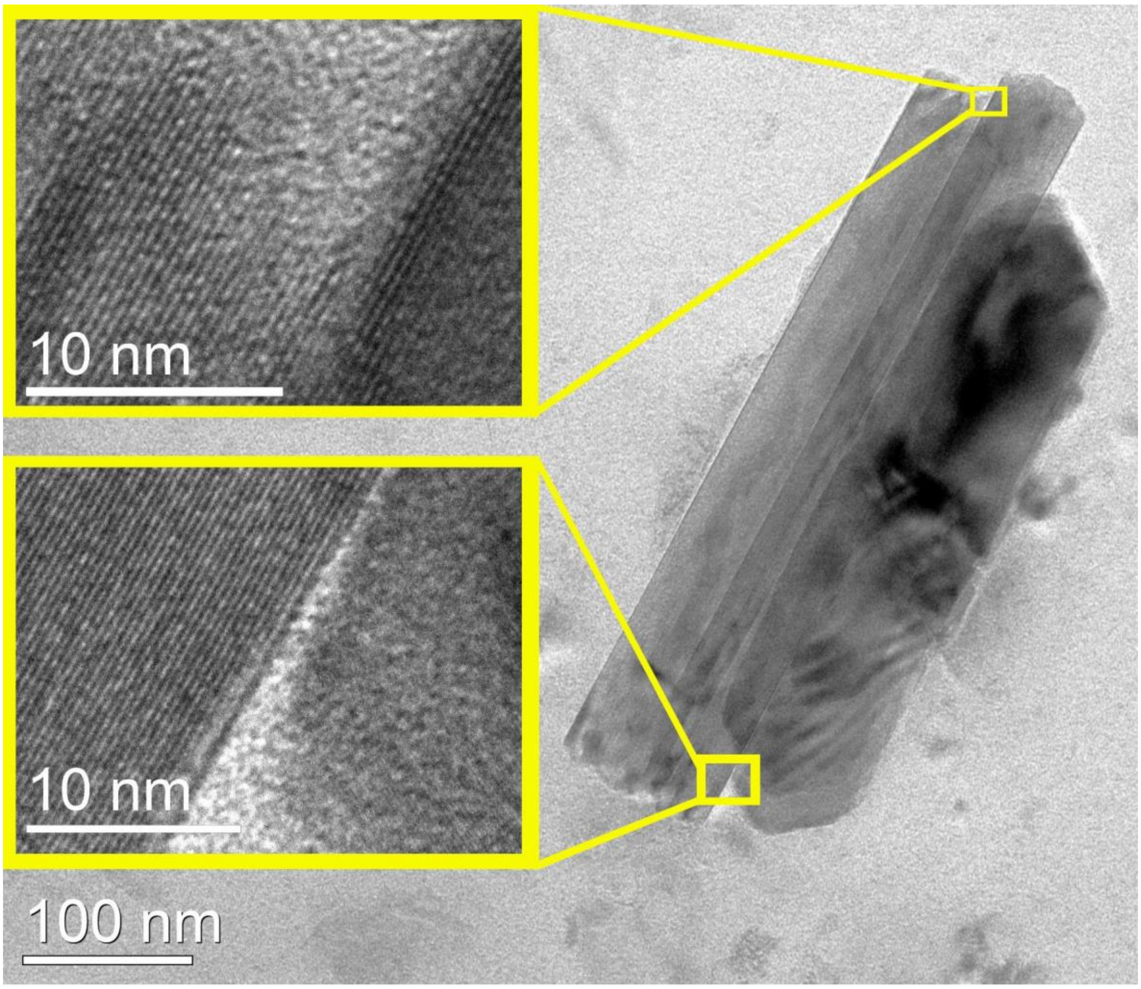

Fig. S32: HR-TEM showing presence of microcrystals for D-Ile-L-Phe, as confirmed by the presence of diffraction lines (insets), in agreement with optical microscopy data.

10. CryoTEM imaging data

Freshly prepared samples were vitrified with Vitrobot Mark IV (Thermo Fisher Scientific, Waltham, MA, US). Protochips C-Flat 2/2, 200 mesh copper EM grids (Protochips, Morrisville, NC, USA) were glow discharged for 60 s at 20 mA and positive polarity in air atmosphere (GloQube® Plus, Quorum, Laughton, UK). Vitrobot conditions were set to 22 °C, 80 % relative humidity, blot time: 6 s and blot force: 2. 3 µl of the sample suspension was applied to the grid, blotted and immediately vitrified in liquid ethane. Samples were visualized under cryo conditions with the 200 kV microscope Glacios equipped with the Falcon 3EC detector (Thermo Fisher Scientific, Waltham, MA, US).

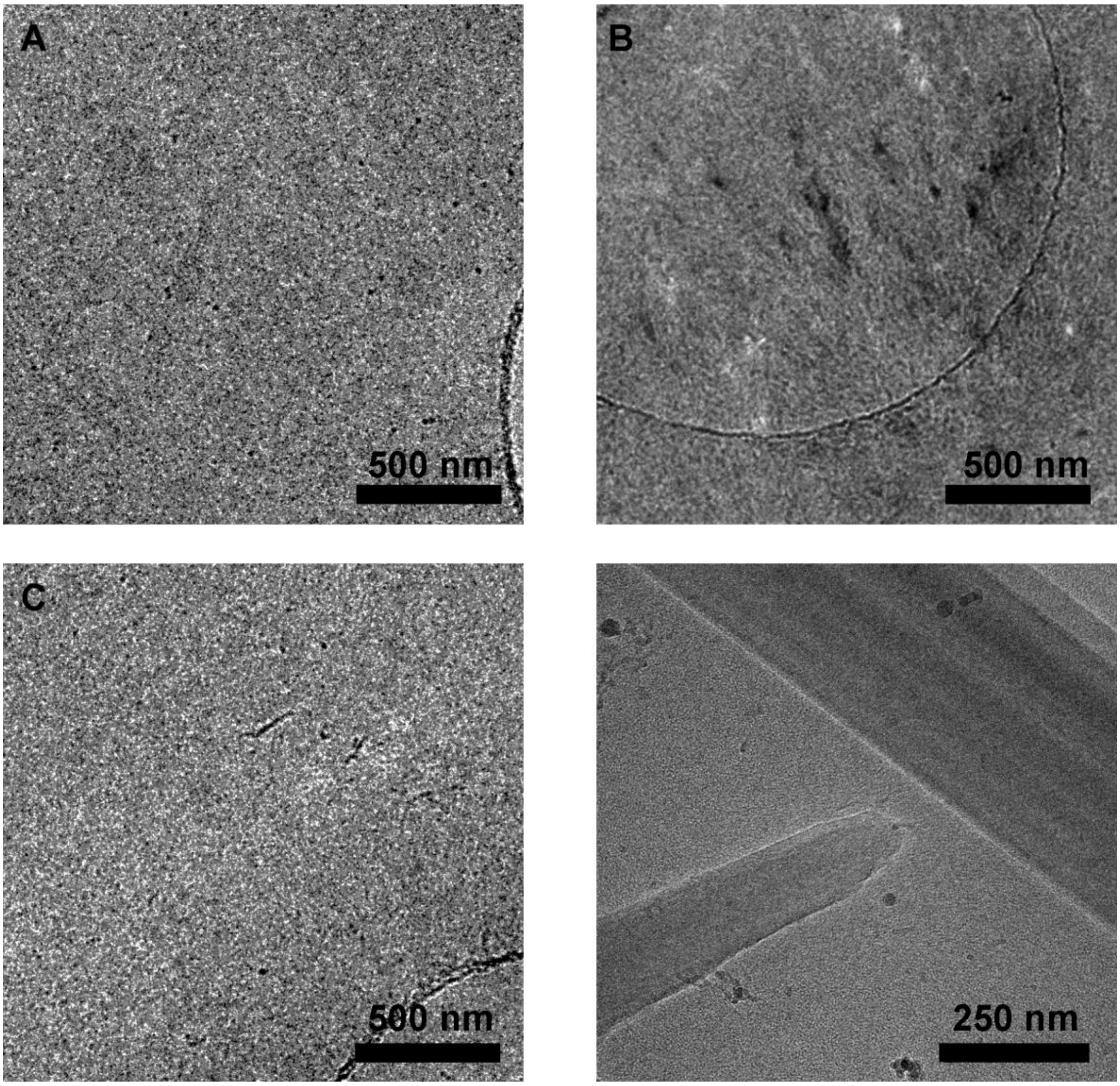

Fig. S33: CryoTEM micrographs of (**A**) L-Ile-L-Phe, (**B**) D-Ile-L-Phe, (**C**) L-Phe-L-Ile, and (**D**) D-Phe-L-Ile.

11. ^{19}F-NMR, TFA removal and self-assembly with chloride counterion

2.1 mg of peptide D-Phe-L-Ile was dissolved in DMSO-d_6 (0.75 mL). 8 µl of internal standard α,α,α-trifluorotoluene (Sigma-Aldrich T63703-500G, Lot# S45042V CAS:98-08-8) were added to the peptide solution to have a 1:8 of peptide to internal standard.

Then, 4.0 mg of peptide were dissolved in millliQ water (1.0 mg/mL) and 104 µL of a 100 mM solution of HCl were added to remove the trifluoroacetate (TFA) peptide counterion and exchange it with chloride. The solution was mixed by vortexing and sonication and left at room temperature for two minutes. The solution was lyophilized overnight and the peptide powder was analyzed again by 400 MHz (DMSO-d_6, TMS) as above.

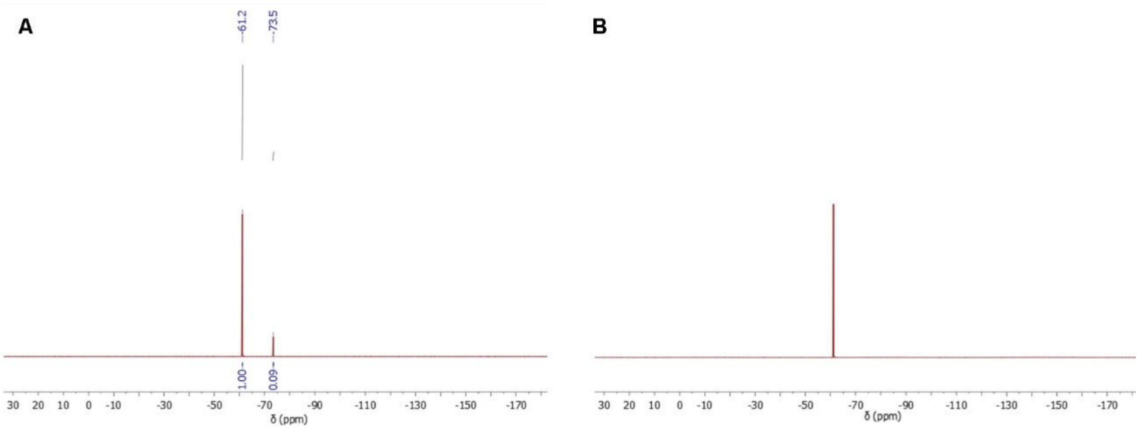

Fig. S34. ^{19}F-NMR of the gelator with the internal standard allowed for TFA quantification (A), and confirmation of TFA removal (B) after exchange with chloride counterion following the procedure described above.

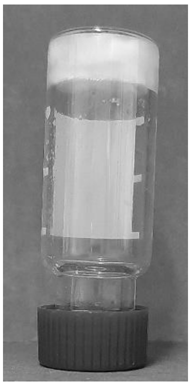

Fig. S35. Photograph of the gel of D-Phe-L-Ile (40 mM) with chloride counterion in PBS.

12. Self-assembly in 10 mM PBS and deionized water

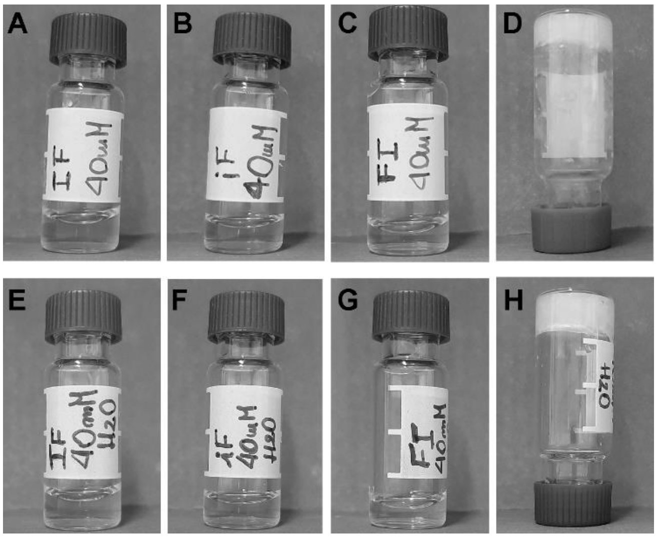

Fig. S36. Photographs of self-assembly tests in 10 mM PBS (A-D) or deionized water adjusted to the same pH (E-H) for each dipeptide at 40 mM: (**A, E**) L-Ile-L-Phe, (**B, F**) D-Ile-L-Phe, (**C, G**) L-Phe-L-Ile, and (**D, H**) D-Phe-L-Ile.
